# Pyrroloquinoline Quinone Reprograms the Single‐Cell Landscape of Immune Aging in Hematopoietic Immune System

**DOI:** 10.1111/acel.70050

**Published:** 2025-04-07

**Authors:** Xiuxing Liu, Chun Zhang, Jianjie Lv, Yidan Liu, Chenyang Gu, Yuehan Gao, Wen Ding, Hui Chen, Nanwei Xu, Hongbin Yin, Wenru Su, Zhuping Xu

**Affiliations:** ^1^ Department of Ophthalmology West China Hospital, Sichuan University Chengdu Sichuan China; ^2^ State Key Laboratory of Ophthalmology Zhongshan Ophthalmic Center, Sun Yat‐sen University, Guangdong Provincial Key Laboratory of Ophthalmology and Visual Science Guangzhou Guangdong China; ^3^ Guangzhou Women and Children‘s Medical Center Guangzhou Medical University Guangzhou Guangdong China; ^4^ Clinical Medicine (Eight‐Year Program) West China School of Medicine, Sichuan University Chengdu Sichuan China; ^5^ Department of Ophthalmology Ninth People‘s Hospital, Shanghai JiaoTong University School of Medicine Shanghai China

**Keywords:** aging, hematopoietic immune system, oxidative stress, pyrroloquinoline quinone, senescent cells

## Abstract

Aging is an inevitable biological process, driven in part by increased oxidative stress, which accelerates cellular damage and contributes to immune system dysfunction. Therefore, targeting oxidative stress has emerged as a potential strategy. Pyrroloquinoline quinone (PQQ), a potent antioxidant, has demonstrated significant efficacy in reducing oxidative stress and modulating immune responses, making it a promising therapeutic candidate. In this study, we investigated the effects of aging on the hematopoietic immune system (HIS) through single‐cell RNA sequencing (scRNA‐seq) of spleen and bone marrow cells in murine models. Our results revealed widespread age‐related inflammation and oxidative stress within immune cell populations. Notably, long‐term PQQ supplementation improved physiological parameters and reduced blood inflammatory factors levels in aged mice. Subsequent scRNA‐seq analysis demonstrated that PQQ supplementation effectively reduced oxidative stress levels across various HIS cell types and reversed aging‐related phenotypes, such as inflammatory responses and immunosenescence. Additionally, PQQ reversed aging‐induced disrupted signaling and restored immune homeostasis, particularly in B cells and hematopoietic stem cells (HSCs). Importantly, we identified critical molecular targets, including ASPP1, which mediates PQQ's anti‐apoptotic effects in B cells, and Yy1 and CD62L, which were upregulated by PQQ to restore HSCs self‐renewal and differentiation potential. Furthermore, the machine learning program and experimental validation demonstrated the senolytic and senomorphic effects of PQQ in vivo and vitro. These findings underscore PQQ's potential not only in mitigating oxidative stress but also in restoring immune homeostasis and promoting cellular regeneration, highlighting its therapeutic potential in addressing immune aging and improving physiological function.

## Graphical abstract

This study demonstrates that aging induces oxidative stress and inflammation in the hematopoietic immune system, resulting in immune homeostasis dysfunction, including alterations in B cells and hematopoietic stem cells, senescent cells (SnCs) accumulation, and senescence‐associated secretory phenotypes (SASPs) production. Notably, the study highlights that Pyrroloquinoline Quinone (PQQ), a potent antioxidant, exerts both senolytic and senomorphic effects, effectively reducing oxidative stress and immunosenescence, underscoring its potential for mitigating age‐related immune dysfunction.

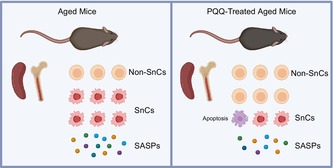

## 1 Introduction

Aging is a universal, gradual, and irreversible biological process that occurs in cells, tissues, and organs (Luo et al. 2022). Over the past 200 years, life expectancy has risen due to medical advances, along with the emergence of medical and social challenges related to aging. It is characterized by a progressive loss of physiological integrity, including the immune system, leading to impaired function and increased vulnerability to mortality (López‐Otín et al. 2013; Mogilenko et al. 2022). Cellular senescence serves as a key indicator of biological aging (López‐Otín et al. 2023). The accumulation of senescent cells (SnCs) has been linked to numerous non‐proliferative diseases, including kidney disease, pulmonary fibrosis, atherosclerosis, and neurodegenerative disorders such as Alzheimer's disease, ultimately contributing to a heightened risk of mortality (López‐Otín et al. 2023). In summary, aging significantly impacts physiological functions and is associated with susceptibility to a range of diseases.

The immune system plays a crucial role in maintaining homeostasis (Antonangeli et al. 2021). As aging occurs, it undergoes a series of age‐related changes that impact its ability to respond to new challenges. Aging alters the composition and function of various immune cells, including B cells (bc), T cells, and myeloid cells (Nikolich‐Žugich 2018). This is mainly manifested by a decrease in naive cells and an increase in effector and exhausted cells (Mogilenko et al. 2021). This phenomenon, often referred to as immunosenescence, heightens the body's susceptibility to tumors, infectious diseases, and neurodegenerative disorders (Mogilenko et al. 2022). For instance, in the case of stroke, aging leads to neutrophil (NEU) dysfunction, resulting in the rapid accumulation of pro‐inflammatory NEU in the bloodstream and ischemic brain, which exacerbates the condition (Gullotta et al. 2023). The spleen and bone marrow are key organs of the hematopoietic and immune systems (HIS), containing a complete range of immune cell components at different stages of development and playing a vital role in the body's immune response (Haas et al. 2018; Zhao et al. 2012). We have previously reported significant effects of aging on these hematopoietic immune organs, including increased levels of senescence‐associated secretory phenotype (SASP) factors in various immune cells, as well as changes in stem cell damage and differentiation deviations (Lv et al. 2024). However, the mechanisms driving these age‐related changes in HIS still need further investigation to offer valuable insights for reversing the aging process.

Over the past century, it has become evident that the incidence of age‐related diseases is closely linked to elevated oxidative stress with age. Oxidative stress can induce both apoptosis and necrosis of cells, thereby impairing healthy aging (Vatner et al. 2020). More importantly, oxidative stress significantly affects the longevity, self‐renewal, and differentiation of hematopoietic stem cells (HSCs) (Morales‐Hernández et al. 2018). HSCs with high levels of reactive oxygen species (ROS) exhibit decreased self‐renewal capacity and a bias toward myeloid differentiation, while those with low ROS levels demonstrate enhanced self‐renewal ability and sustained transplantation potential (Jang and Sharkis 2007). Pyrroloquinoline quinone (PQQ) is an aromatic tricyclic o‐quinone that was originally identified as a coenzyme of methanol dehydrogenase (Geng et al. 2019). In its reduced form, PQQ exhibits antioxidant activity that is seven times more effective at scavenging free radicals than vitamin C (Akagawa et al. 2016). As a powerful antioxidant, PQQ has been shown in previous studies to play a beneficial role in age‐related diseases such as intervertebral disk degeneration (Xue et al. 2024), diabetic nephropathy (Zhang, Zhang et al. 2020), osteoporosis (Li et al. 2023), cognitive deficits (Ohwada et al. 2008), and the prevention of osteoarthritis (Qin et al. 2019), primarily by inhibiting oxidative stress. Additionally, PQQ alters the composition and proportion of T cells in models of allergic inflammation, alleviating allergic airway inflammation by modulating the JAK–STAT signaling pathway (Min et al. 2022). In clinical trials, supplementation with 0.2 and 0.3 mg/kg of PQQ has been shown to effectively enhance the body's antioxidant capacity and reduce systemic inflammation in human subjects (Harris et al. 2013). These findings suggest that PQQ may serve as a potential therapeutic agent for mitigating inflammatory damage and age‐related diseases while also playing a crucial role in regulating the immune microenvironment. However, there is a lack of study investigating whether the application of PQQ can improve the aging of the HSC.

In this study, we observed a significant increase in oxidative stress during the HIS aging process, while long‐term treatment with the antioxidant PQQ provided notable geroprotective effects by exerting antioxidant, anti‐aging, and anti‐inflammatory properties. Specifically, PQQ reduced oxidative stress‐induced senescence processes in BC and restored aging‐induced alterations in differentiation and proliferation of HSCs. Furthermore, our identification of SnCs using a machine learning program revealed a close association with oxidative stress, and PQQ demonstrated senolytic activity and anti‐inflammatory effects on SnCs. These findings suggest that PQQ not only mitigates aging‐associated immune dysfunction but also exhibits significant geroprotective and regenerative effects, offering a novel therapeutic approach to counteract immune system decline associated with aging.

## 2 Results

### 2.1 Aging‐Induced Changes in Gene Expression and Biological Processes in HIS

**FIGURE 1:**
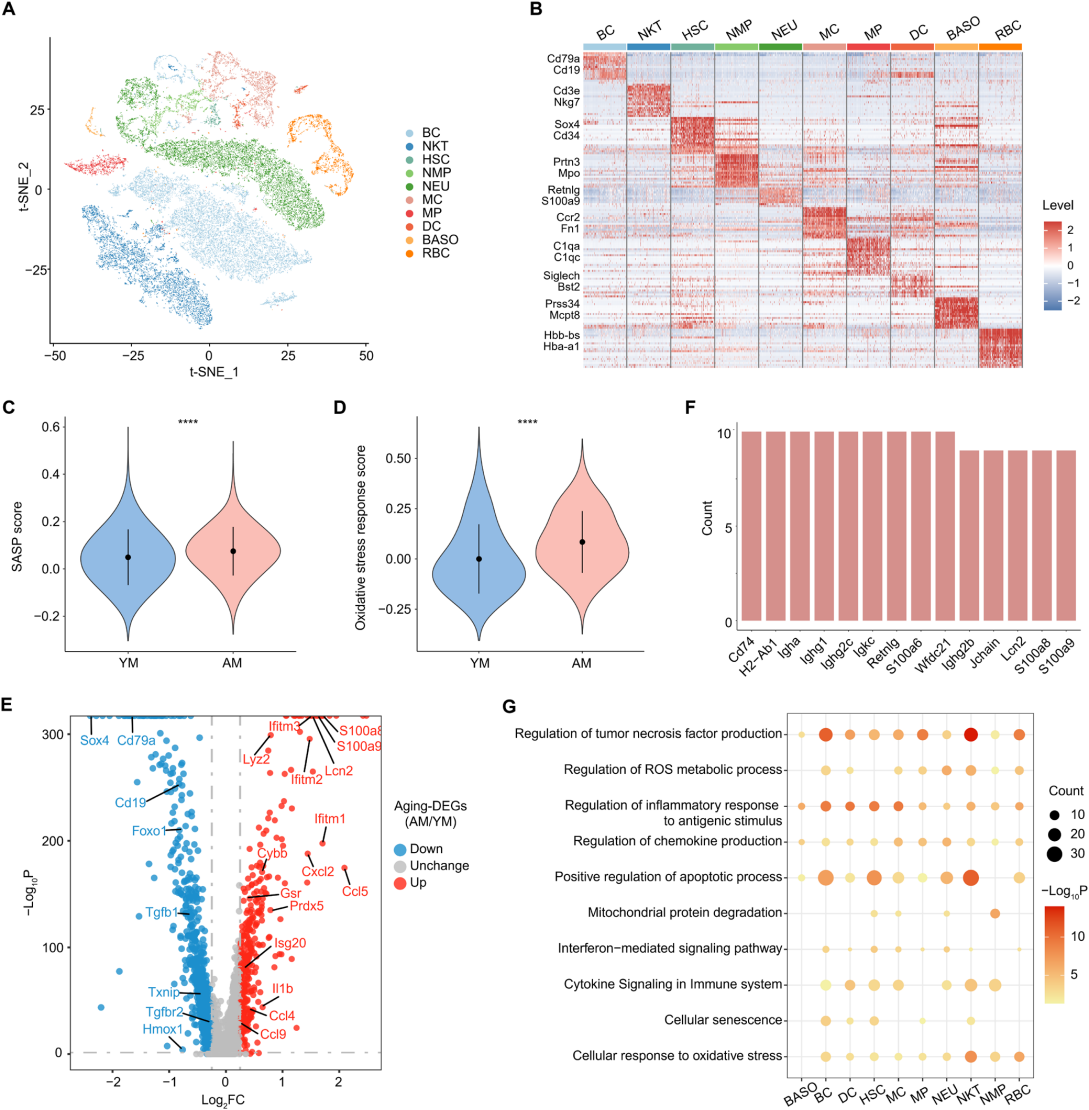
The scRNA‐seq analysis on HIS cells of the YM and AM groups. (A) t‐SNE plot showing the immune cell subpopulations of HIS in scRNA‐seq. (B) Heatmap showing scaled expression of discriminative gene sets for each cell subpopulation. (C, D) Violin plots showing the score of SASP (C) and oxidative stress response (D) between the two groups. (E) Volcano plot showing the up‐ or down‐regulated aging‐DEGs in overall HIS cells. (F) Bar chart showing the frequencies of the upregulated aging‐DEGs observed across 10 or 9 immune cell subpopulations. (G) Representative GO biological processes and pathways enriched in upregulated aging‐DEGs in immune cell subpopulations of HIS. Data are shown as mean ± SD. *p* were analyzed using Student's t‐test (C, D); ****p < 0.0001.

To explore the impact of aging on systemic immunity, we analyzed scRNA‐seq data of spleen and bone marrow cells from young mice (YM, 2–3 weeks old) and aged mice (AM, 19–21 months old), respectively. After quality control, cell data were dimensionally reduced using t‐SNE and divided into 10 groups according to their specific markers, which were bc, natural killer and T cell (NKT), HSC, neutrophil‐myeloid progenitor (NMP), NEU, monocyte (MC), macrophage (MP), dendritic cell (DC), basophil (BASO) and red blood cell (RBC) (Figure 1A,B).

To investigate the transcriptional impact of aging on HIS cells, we assessed biological scores associated with the aging process to evaluate the effects of natural aging and intercellular differences. We found that the SASP and oxidative stress levels in HIS immune cells were significantly increased after aging (Figure 1C,D). In addition, we also performed inflammatory response and cell division scores among the groups, which suggested that aging caused increased inflammatory response and decreased cell division ability (Figure S1A,B). These results are consistent with previously reported studies, validating the reliability of our methods.

We next performed differentially expressed genes (DEGs) analysis between the two groups. The results showed that inflammation‐related genes (such as S100a family, interferon family and CXCL2) were upregulated in the AM group, while developmental regulation and homeostasis‐related genes (Tgfb1, Tgfbr2, Txnip, Hmox1, Foxo1, Sox4) were downregulated (Figure 1E). The downregulation of Tgf‐related genes is consistent with a previous study on the aged HSC transcriptome (D. Sun et al. 2014). We also investigated the upregulated aging‐DEGs among cell subpopulations and found that a large number of genes related to inflammation and autoimmune response were upregulated in nearly all types (Figure 1F). To further clarify the specific biological processes involved in upregulated aging‐DEGs, we performed GO analysis and found that they were mainly enriched in several processes related to inflammatory response, oxidative stress, and apoptosis‐related pathways (Figure 1G). Among them, bc, HSC, NEU, and NKT were more significantly enriched in the above biological processes, suggesting that they may be more affected by aging. In general, aging induces widespread changes in the immune system, with inflammation and oxidative stress being the main processes involved.

### 2.2 PQQ Ameliorates Aging‐Induced Changes in the Body and Immune System

**FIGURE 2:**
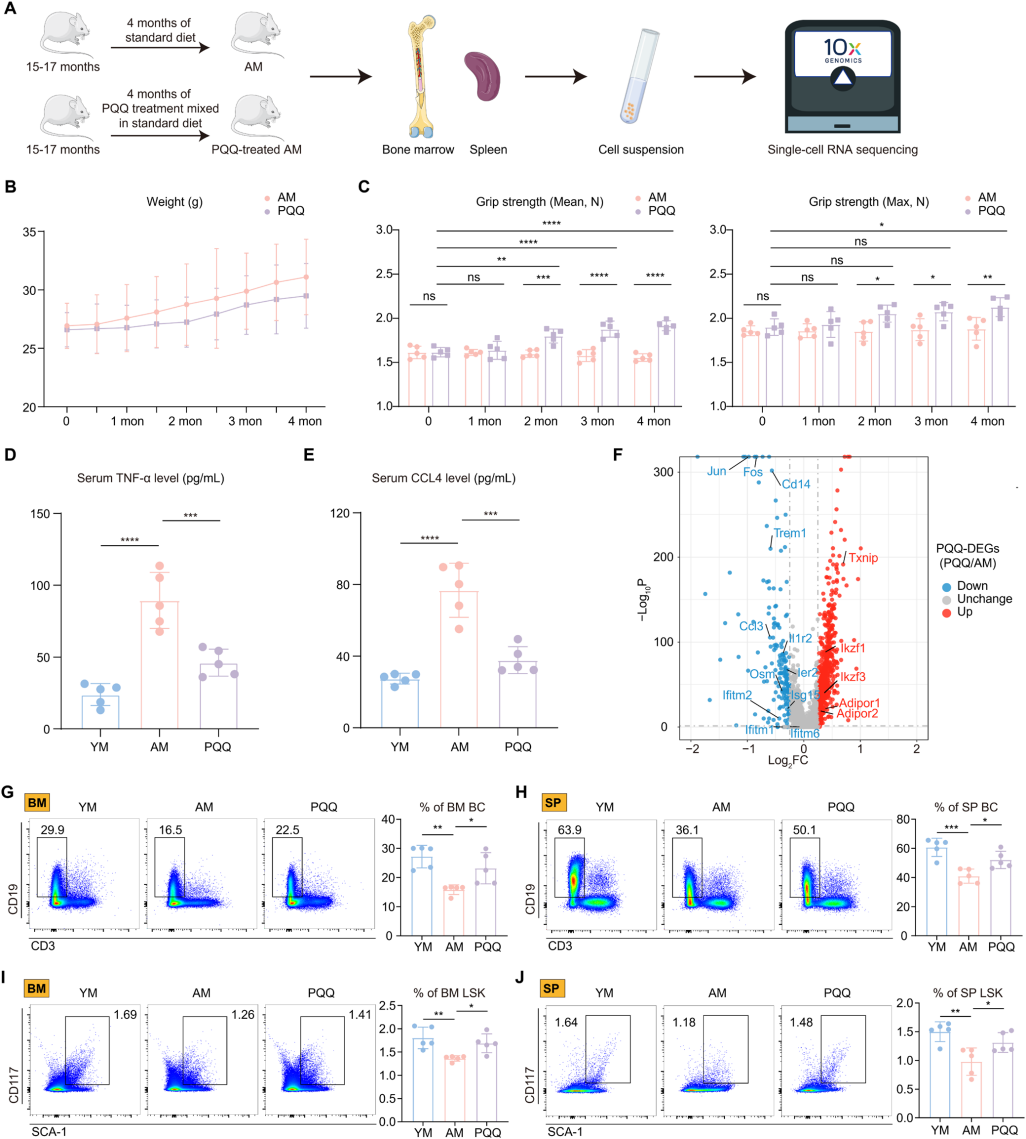
The effects of PQQ on physiological index and HIS cells. (A) Schematic diagram of experimental design for standard diet or PQQ‐added diet and following scRNA‐seq analysis of HIS cells from AM group and PQQ‐treated AM group. (B) Line graph showing the mouse weight (g) during the 4 months of experiments (*n* = 5/group). (C) The column charts showing the mean grip strength (left) and max grip strength (right) between the two groups during the 4 months of experiments (*n* = 5/group). (D, E) The column charts show the serum levels of TNF‐α (D) and CCL4 (E) among the three groups. (F) volcano plot showing the up‐ or down‐regulated PQQ‐DEGs in overall HIS cells. (G–J) The FCM histograms (left) and column charts (right) show the percentage of BM BC (G), SP BC (H), BM LSK (I), and SP LSK (J) among the three groups (*n* = 5/group). Data are shown as mean ± SD. *p* were analyzed using two‐way ANOVA (C) or one‐way ANOVA (D, E, G‐J); ns, non‐significant, *p < 0.05, **p < 0.01, ***p < 0.001, ****p < 0.0001.

Oxidative stress, as a key process identified above in which aging affects the immune system, its inhibition is emerging as a potential mechanism for delaying and even reversing aging changes (Vatner et al. 2020). PQQ, a redox cofactor, has excellent antioxidant properties that can help scavenge free radicals and reduce cellular damage, which has led to its positive role in cardiovascular health and metabolic regulation (Jonscher et al. 2021). Therefore, we added PQQ to the standard feeding of 15–17‐month mice and fed continuously for 4 months, after which spleen and bone marrow cells were taken for scRNA‐seq (Figure 2A). During the 4 months of standard and PQQ‐supplemented feeding, we weighed both groups of mice and assessed their muscle strength. The results indicated that PQQ effectively controlled body weight and enhanced muscle strength in the AM group (Figure 2B,C). Additionally, we employed an enzyme‐linked immunosorbent assay (ELISA) to measure the serum levels of the inflammatory cytokine TNF‐α and the chemokine CCL4. These circulating SASP factors were significantly elevated in the AM group but showed a general decline following long‐term PQQ treatment (Figure 2D,E).

To further explore the effects of PQQ on HIS aging, we performed DEGs analysis between the PQQ and AM groups. The volcano plot showed that PQQ downregulated oxidative stress and inflammatory response‐related genes (Jun, Fos, and Trem1) and upregulated immune regulation‐related genes (Txnip, Ikzf1, and Ikzf3) (Figure 2F). In addition, PQQ increased the levels of adiponectin receptors (Adipor1, Adipor2), which activate downstream pathways to exert geroprotective effects (Iwabu et al. 2015). We next performed SASP and oxidative stress scores on these two groups, suggesting that SASP and oxidative stress levels were decreased in PQQ‐supplemented AM, consistent with their antioxidant effects (Figure S2A,B). Also, the aging‐induced increase of inflammatory response and decrease of cell division capacity were reversed by PQQ (Figure S2C,D). After that, based on scRNA‐seq data, we calculated the proportions of immune cells in the YM, AM, and PQQ groups, respectively, and found that the decreased bc, HSC proportions were significantly restored after supplementation of PQQ (Figure S2E). Finally, we conducted flow cytometry (FCM) and observed a reduction in the frequency of bc in BM and SP following aging. However, these populations increased following PQQ treatment (Figures 2G,H and S2F). Similar results were also found in the ratio of Lin‐ SCA‐1+ CD117+ LSK in BM and SP (Figure 2I,J and S2G).

In general, PQQ supplementation has demonstrated the capacity to partially reverse age‐related physiological changes and mitigate oxidative stress and inflammatory responses in HIS aging.

### 2.3 PQQ Reverses Aging‐Related Gene Expression Changes in the Immune System

**FIGURE 3:**
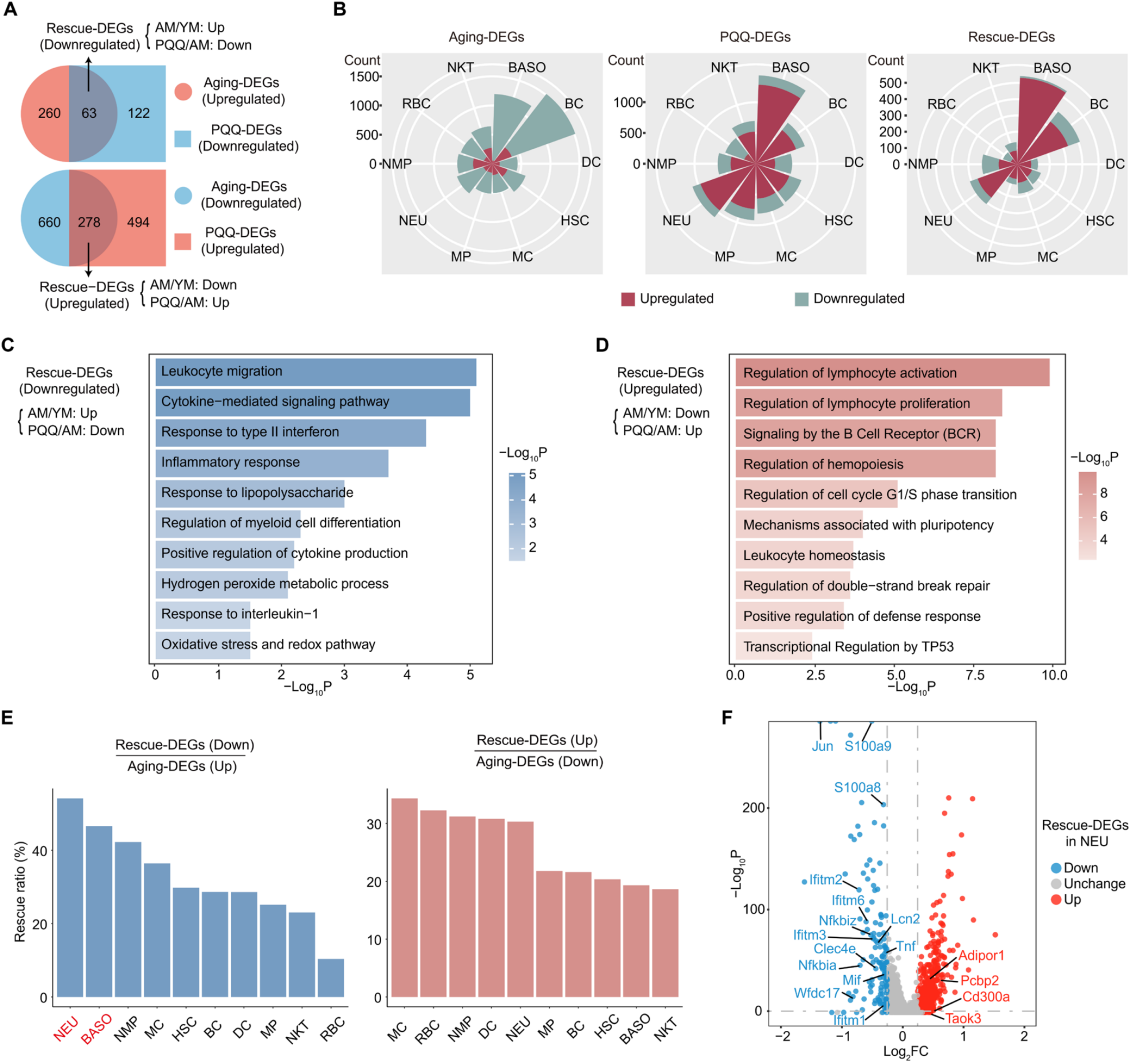
PQQ reverses aging‐related gene expression changes in HIS cells. (A) Venn diagram showing the interaction (up) of upregulated aging‐DEGs and downregulated PQQ‐DEGs and that (down) of downregulated aging‐DEGs and upregulated PQQ‐DEGs. The black arrows indicate downregulated (up) or upregulated (down) rescue‐DEGs. (B) Rose plots showing the count of upregulated and downregulated aging‐, PQQ‐, or rescue‐DEGs among cell subpopulations. (C, D) Representative GO biological processes and pathways enriched in downregulated (C) and upregulated (D) rescue‐DEGs in HIS cells. (E) Bar chart showing the ratio of rescue‐DEGs to aging‐DEGs in each cell subpopulation. (F) Volcano plot showing the up‐ or down‐regulated rescue‐DEGs in NEU subset.

To reveal the molecular events associated with PQQ's reversal of immune system aging, we identified the DEGs between AM and YM mice as well as between PQQ‐added‐standard‐fed AM and standard‐fed AM mice and termed them “aging‐DEGs” and “PQQ‐DEGs” respectively. Further integrative comparative analysis of these DEGs identified aging‐DEGs that were partially rescued by PQQ and termed them “rescue‐DEGs” (Figure 3A). Next, to distinguish the effects of aging and PQQ on each immune cell subtype, we calculated the number of aging‐, PQQ‐, and rescue‐DEGs for each cell subtype separately. Notably, as shown in the rose diagram, bc, BASO, and HSC were the immune cell subtypes mainly affected by aging, whereas BASO, NEU, and MP were the immune cell subtypes mainly affected by PQQ. Rose diagrams based on rescue‐DEGs further demonstrated that PQQ remarkably reversed gene expression changes in BASO and bc affected by aging (Figure 3B). These analyses highlight the cell effects of PQQ on HIS aging.

Subsequently, we explored the biological effects of aging‐, PQQ‐, and rescue‐DEGs on immune cells by performing GO and pathway analyses. The results showed that upregulated aging‐DEGs were mainly enriched in oxidative stress and inflammatory response‐related processes, which were antagonized by PQQ (Figure S3A,B). Aging also led to a synchronized decrease in multiple pathways involved in lymphocyte activation, lymphocyte proliferation, and leukocyte homeostasis, which was reversed by PQQ (Figure S3C,D). GO and pathway analyses of the rescue‐DEGs further supported the role of PQQ in anti‐inflammatory, anti‐oxidative stress, regulation of immune system homeostasis, and the cell cycle, all of which contribute to immune system aging protection (Figure 3C,D). Next, we measured the mRNA levels of several aging and SASP‐related genes using RT‐qPCR. The analysis revealed that Cdkn2a (encoding p16), Cdkn1a (encoding p21), and several SASP factors were elevated in aged HIS cells but decreased following PQQ treatment (Figure S3E). These findings suggest that PQQ not only suppresses aging‐related markers but also reverses certain signaling mechanisms disrupted by aging, which are crucial for maintaining normal immune homeostasis.

We further investigated the cell specificity of PQQ's rescue effect based on the ratio of rescue‐DEGs to aging‐DEGs (Figure 3E). NEU and BASO, which were the two cell types with the highest rescue ratio of downregulated rescue‐DEGs, were restored to homeostasis most efficiently by PQQ. PQQ efficiently reversed the expression of more than 40% of the upregulated aging‐DEGs in these cells (Figure 3E). Therefore, we further analyzed the rescue‐DEGs in these cell subpopulations. In NEU, aging induced upregulation of inflammatory genes (such as S100a family and interferon family) and reduced the expression of anti‐inflammatory genes (Pcbp2, Cd300a, Taok3) and adiponectin receptor (Adipor1), which were reversed by PQQ (Figure 3F). Moreover, in BASO, PQQ also rescued the decrease of anti‐inflammatory genes (Ncor1, Taok3, Pten, Foxp1, Cd200r1) and sirtuins (Sirt3, Sirt7), as well as the increase of genes associated with inflammatory response (Figure S3F). Collectively, PQQ counteracts aging by inhibiting oxidative stress and inflammatory response pathways, while simultaneously promoting the expression of immunomodulatory genes in a cell‐specific pattern.

### 2.4 PQQ Exerts Anti‐Apoptotic Effects Through ASPP1 to Rescue Aging‐Induced B Cell Changes

**FIGURE 4:**
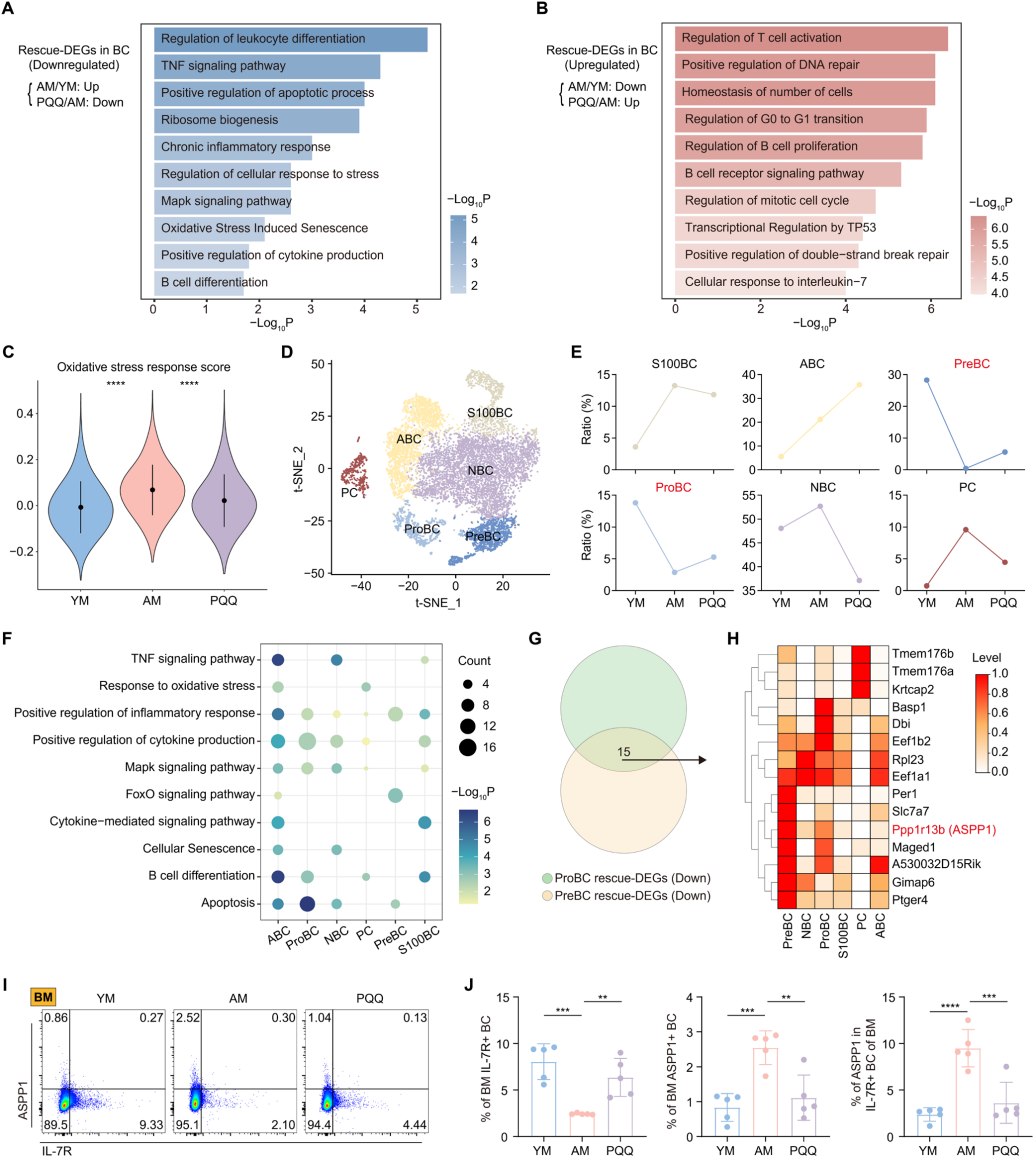
The effects of PQQ on BC. (A, B) Representative GO biological processes and pathways enriched in downregulated (A) and upregulated (B) rescue‐DEGs in BC. (C) Violin plots showing the score of oxidative stress response among the three groups. (D) t‐SNE plot showing the BC subpopulations in scRNA‐seq. (E) Line graph showing the ratio of BC subsets among the three groups. (F) Representative GO biological processes and pathways enriched in downregulated rescue‐DEGs among BC subsets. (G) Venn diagram showing the interaction of downregulated rescue‐DEGs in ProBC and PreBC. (H) Heatmap showing scaled expression of 15 genes identified in G among BC subsets. (I,J) The FCM histograms show the percentage of IL‐7R (I) and ASPP1 (J) in BM BC among the three groups. The column charts showing the percentage of BM IL‐7R+ BC (left), BM ASPP1+ BC (mid), and ASPP1 in BM IL‐7R+ BC (right) among the three groups (*n* = 5/group). Data are shown as mean ± SD. *p* were analyzed using one‐way ANOVA (C–J); **p < 0.01, ***p < 0.001, ****p < 0.0001.

B cells are a key cell subtype involved in immune responses, and their function is affected by aging (Frasca et al. 2020). We have showed that PQQ could reverse the decline in BC proportions caused by aging. Therefore, we explored the effects of PQQ on changes in the subsets and biological processes caused by aging. We performed GO and functional enrichment analysis on the rescue‐DEGs in BC, suggesting that PQQ mainly inhibited aging‐induced inflammatory, apoptotic, and oxidative stress processes, such as the TNF signaling pathway, positive regulation of apoptotic process, and oxidative stress‐induced senescence (Figure 4A). The upregulated rescue‐DEGs were mainly enriched in processes related to cell homeostasis and cycle regulation (Figure 4B). Additionally, PQQ upregulated BC functions and immune response‐related processes that are diminished with aging, including the regulation of T cell activation, the B cell receptor signaling pathway, and the cellular response to interleukin‐7. Subsequently, we assessed oxidative stress scores in the three groups of BC, revealing that PQQ's antioxidant capacity effectively mitigated the aging‐induced oxidative stress levels in BC (Figure 4C). The protective effects of PQQ against age‐related changes in BC were also evident in the scores for SASP, inflammatory response, and cell division (Figure S4A–C).

To further clarify the effect of PQQ on different BC subtypes, we divided bc into 6 groups based on classic markers, including precursor B cells (PreBC), naïve B cells (NBC), proliferating B cells (ProBC), S100A+ B cells (S100BC), plasma cells (PC), and age‐associated B cells (ABC) (Figure 4D, Figure S4D). The ratio analysis demonstrated the restorative effects of PQQ supplementation on the aging‐altered BC landscape, revealing that the age‐related reductions in Prebc and ProBC ratios were effectively reversed by PQQ treatment (Figure 4E). Then, we investigated the effects of PQQ on aging‐induced alterations in gene expression within BC subsets, suggesting that PQQ mainly reversed the upregulated and downregulated aging‐DEGs in PreBC and ProBC (Figure S4E). We conducted GO and functional analyses on the downregulated rescue‐DEGs in BC subpopulations and found that PQQ significantly inhibited inflammation, cell differentiation –related biological processes. Specifically, PQQ reduced TNF inflammatory responses in ABC, NBC, and S100BC, cytokine production in ABC, NBC, ProBC, and S100BC, and B cell differentiation in ABC, ProBC, PC, and S100BC. Additionally, consistent with the cell proportion trends, PQQ was able to reverse the increased apoptosis in Probc and PreBC (Figure 4F). We found that ProBC and PreBC cells both had high levels of Il7r (Figure S4F), consistent with previous studies on the key role of IL‐7 signaling in BC regeneration and proliferation (Kaiser et al. 2023; McLean and Mandal 2020).

To identify the potential targets of PQQ, we took the intersection of downregulated rescue‐DEGs of Probc and PreBC and displayed the levels of these 15 intersection genes among bc subtypes (Figure 4G,H). Among them, the level of apoptosis‐related gene Ppp1r13b (encoding ASPP1) (Schittenhelm et al. 2024) in Probc and Prebc was higher than that in other subtypes, suggesting that it may be a potential target for PQQ to exert its anti‐apoptotic effect (Figure 4H). Thus, we performed FCM analysis in BM cells and found that, compared to YM, AM exhibited a decreased proportion of IL‐7R+ bc and an increased expression of the apoptosis‐related marker ASPP1 in bc. PQQ treatment was found to increase the proportion of IL‐7R+ bc while decreasing the proportion of ASPP1+ BC (Figure 4I,J). Notably, the levels of ASPP1 in IL‐7R+ BC were elevated in aged mice and decreased following PQQ treatment (Figure 4I,J). Similar findings were also observed in BC of SP (Figure S4G–H). In summary, these results highlight the role of PQQ in combating oxidative stress, apoptosis, and aging while promoting immune regeneration, thereby contributing to the protection and maintenance of BC homeostasis.

### 2.5 PQQ Reverses Aging‐Induced Alterations in HSCs Through Yy1

**FIGURE 5:**
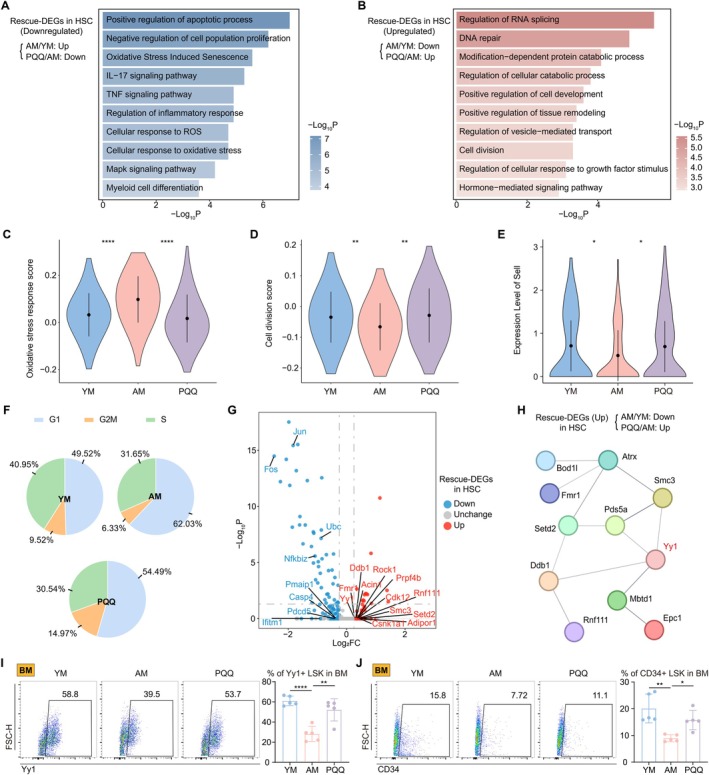
The effects of PQQ on HSCs. (A, B) Representative GO biological processes and pathways enriched in downregulated (A) and upregulated (B) rescue‐DEGs in HSC. (C–E) Violin plots showing the score of oxidative stress response (C), cell division (D), and levels of Sell (E) among the HSCs of three groups. (F) The pie chart shows the proportion of G1, G2M, and S phase in HSC among the three groups. (G) Volcano plot showing the up‐ or down‐regulated rescue‐DEGs in HSC. (H) The gene network showing protein–protein interaction analysis of upregulated rescue‐DEGs in HSC. (I, J) The FCM histograms (left) and column charts (right) show the percentage of BM Yy1+ LSK (I) and BM CD34+ LSK (J) among the three groups (*n* = 5/group). Data are shown as mean ± SD. *p* were analyzed using one‐way ANOVA (C‐E, I, J); *p < 0.05, **p < 0.01, ****p < 0.0001.

HSCs are a type of multipotent stem cell that can differentiate into immune cells, including T, B, and myeloid lineages, so their self‐renewal and differentiation potentials are crucial for maintaining the homeostasis of the immune system. The aging process affects HSCs, leading to a decrease in their functions, and the rescue effect and mechanism of PQQ on these processes remain unclear (Mejia‐Ramirez and Florian 2020). Therefore, we performed GO and functional analysis on the upregulated and downregulated rescue‐DEGs identified in HSCs. Our findings indicated that PQQ countered pathways associated with apoptosis, aging, and oxidative stress in HSCs, while also promoting processes related to damage repair and the regulation of cell development (Figure 5A,B). Our assessment of the scores indicated that PQQ treatment effectively reversed the increase in oxidative stress, SASP, and inflammatory responses in AM, as well as the reduction in cell division (Figure 5C,D, Figure S5A,B).

Next, we investigated the impact of PQQ on the function of aged HSCs, focusing on cell differentiation trends and the cell cycle. Previous studies have reported that aging promotes the myeloid differentiation tendency and reduces the self‐renewal capacity of HSCs (Li et al. 2020). We initially observed that Sell (encoding CD62L), a marker associated with HSC self‐renewal and lineage differentiation, decreased in aged HSCs but increased following PQQ treatment (Figure 5E). We analyzed the differentiation tendency among the three groups of cells and found that PQQ could reverse this myeloid differentiation tendency (Figure S5C). Furthermore, we explored the effect of PQQ on the proliferation ability of HSCs. We evaluated the cell cycle of HSCs, categorizing them into G1, S, and G2/M phases. The results showed that the proportion of the G1 phase in aged HSCs was higher, while the proportion of the S phase was lower compared to young HSCs. PQQ treatment reversed these changes, indicating that PQQ rescued the aging‐induced G1/S phase arrest (Figures 5F and S5D). These findings indicate that PQQ exerts a restorative effect on age‐related alterations in the differentiation and proliferation capacities of HSCs.

Subsequently, we explored the potential targets of PQQ in HSCs. We found that PQQ supplementation reduced the increase in genes related to apoptosis and inflammation induced by aging. Additionally, it upregulated aging‐inhibited adiponectin receptor (Adipor1) and several genes associated with cell proliferation and post‐translational modification (Figure 5G). To further elucidate the functional roles of the proteins encoded by these genes, we conducted protein–protein interaction (PPI) analysis on the upregulated DEGs and identified Yy1 as a core target (Figure 5H). Yy1 is a critical transcriptional regulator implicated in the aging process, and its elevated expression enhances the long‐term engraftment capacity of aged HSCs (Lu et al. 2024; Ma et al. 2022). Accordingly, FCM analysis of BM and SP cells demonstrated that the proportions of Yy1+ LSK were diminished in aged HIS. However, PQQ treatment effectively restored these populations (Figures 5I and S5E). Furthermore, upon induction of Yy1 expression, PQQ was shown to counteract the aging‐related decreases in CD34 and CD62L levels in LSK cells (Figure 5J and S5F–H). This suggests that PQQ may enhance the regenerative potential of HSCs by promoting the expression of key markers associated with stem cell maintenance and function.

Overall, PQQ not only inhibits the classical aging‐related processes but also restores key signaling pathways disrupted by aging, which are crucial for maintaining normal HSCs function and immune homeostasis.

### 2.6 The Program‐Identified SnCs and Experimental Validation Indicate the Senolytic and Senomorphic Properties of PQQ

**FIGURE 6:**
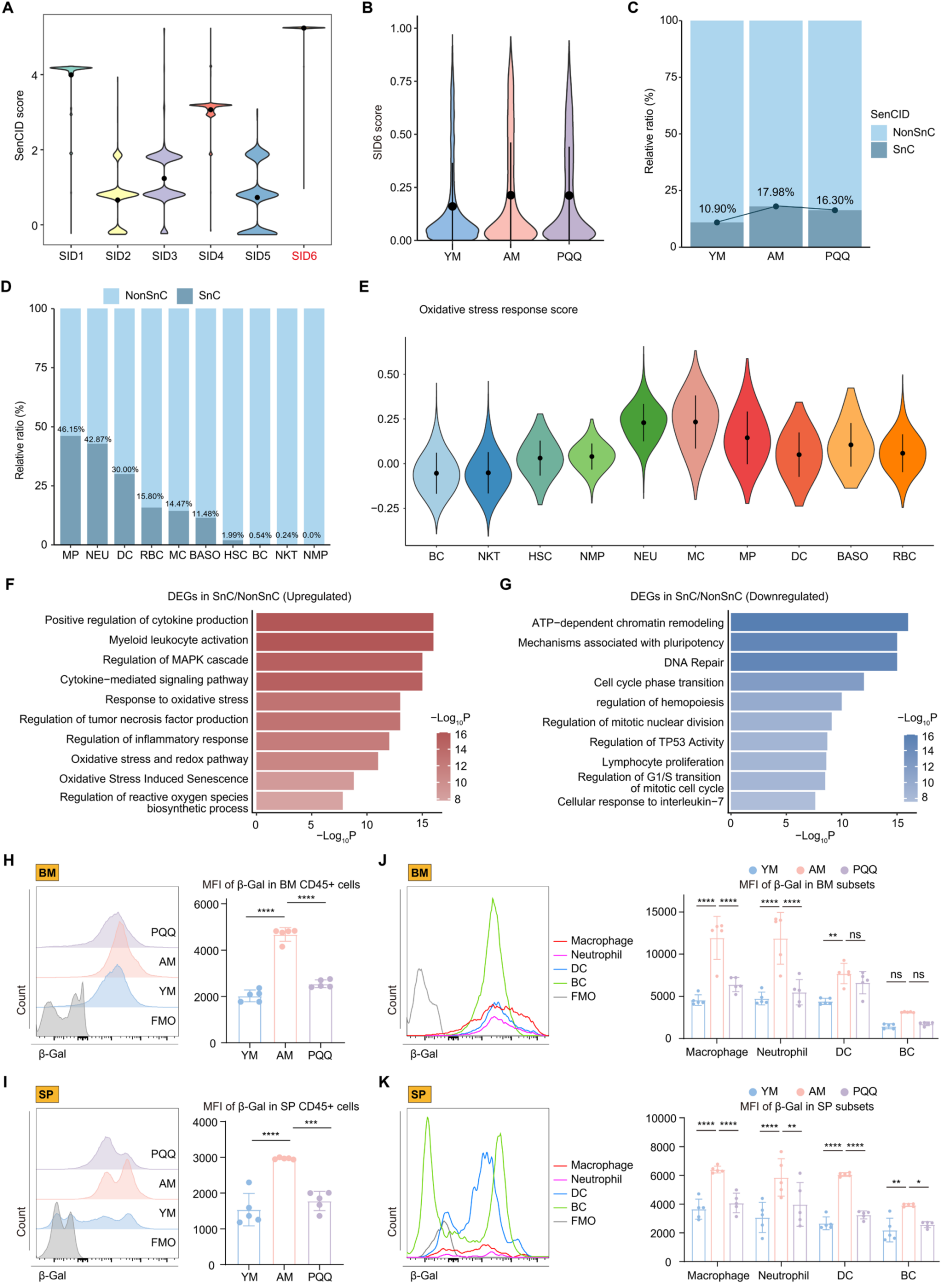
The effects of PQQ on SnCs. (A, B) Violin plots showing the scores of SID1‐6 (A) and the normalized SID6 score (B) among the three groups. (C, D) The column charts show the percentage of SnC cluster and NonSnC cluster among the three groups (C) and cell subpopulations (D). (E) Violin plots showing the score of oxidative stress response among the cell subpopulations. (F, G) Representative GO biological processes and pathways enriched in upregulated (F) and downregulated (G) DEGs in SnC/NonSnC comparison. (H, I) The FCM histograms (left) and column charts (right) showing the MFI of β‐Gal in immune cells of BM (H) and SP (I) among the three groups (*n* = 5/group). (J, K) The FCM histograms (left) and column charts (right) show the MFI of β‐Gal in cell subpopulations of BM (J) and SP (K) among the three groups (*n* = 5/group). Data are shown as mean ± SD. P values were analyzed using one‐way ANOVA (H, I) or two‐way ANOVA (J, K); ns, non‐significant, *p < 0.05, **p < 0.01, ***p < 0.001, ****p < 0.0001.

We have discovered that PQQ effectively counteracts classic age‐related pathways, such as oxidative stress response, SASP, and cell cycle arrest. Following this, we examined whether PQQ affects the clearance of SnCs in aged HIS, based on previous findings that PQQ can reduce SnCs induced by TNF‐α (Hao et al. 2020). To identify SnCs in scRNA‐seq data, we utilized the newly developed and accurate machine learning program, SenCID program (Tao et al. 2024). SenCID detected six key senescence identities (SIDs), and our findings indicated that HIS immune cells were primarily categorized as SID6 according to their scores (Figure 6A). SID6's senescence scores were normalized to a scale of 0 to 1 through logistic regression. We observed an increase in these scores in AM and a decrease in the PQQ group (Figure 6B). Subsequently, the SnC cluster were identified with SenCID using a SID6 score threshold of 0.5, showing a prevalence of 15.53% (Figure S6A). We observed that SnCs accumulated in the aged HIS, and this accumulation was reduced after PQQ treatment (Figure 6C). Upon examining the sources of SnCs, we found that they were primarily made up of myeloid cells, such as MP, NEU, DC, and MC (Figure 6D). Notably, the distribution of oxidative stress response scores among cell populations closely parallels that of SnCs, suggesting a strong correlation between oxidative stress and the presence of SnCs (Figure 6E).

We then explored the functional characteristics of this SnC cluster. Compared to NonSnC, the upregulated DEGs in the SnC cluster were involved in various processes, such as cytokine production, inflammatory responses, myeloid leukocyte activation, and aging‐related processes like oxidative stress response, MAPK signaling, and cell senescence (Figure 6F). In contrast, the SnC cluster exhibited significant reductions in metabolic pathways, cell cycle progression, DNA damage repair mechanisms, and lymphocyte proliferation (Figure 6G). Therefore, PQQ might clear SnCs and reverse age‐related abnormalities. To validate this, we performed FCM experiments using β‐Gal to identify SnCs in vivo and co‐labeled them with specific surface markers for MP (CD11B+ F4/80+), NEU (LY6C^med^CD11B+ LY6G+), DC (CD11C+), and bc (CD19+). We found that β‐Gal+ SnCs were elevated in BM and SP of the AM group but decreased after PQQ treatment (Figures 6H,I and S6B), which matched the trends observed in scRNA‐seq. Additionally, MP and NEU showed high β‐Gal expression, followed by DC and bc (Figure 6J–K), consistent with scRNA‐seq predictions (Figure 6D).

We further investigated the molecular basis underlying the senolytic role of PQQ. The upregulated PQQ‐DEGs in the SnC cluster were significantly associated with apoptotic signaling, death receptor pathways, and programmed cell death, which align with the mechanisms of other senolytics (Kirkland and Tchkonia 2020). Furthermore, PQQ also positively affected normal cellular processes related to cytoskeleton organization, DNA repair, and cell morphogenesis (Figure S6C). Moreover, we observed that within the identified SnC cluster, the oxidative stress response was higher in the AM group compared to the YM group. Notably, PQQ treatment was effective in reducing this oxidative stress response (Figure S6D). It has been reported that the oxidative stress response can induce the formation of SnCs and their secretion of SASP factors, which mediate inflammatory responses (Papaconstantinou 2019). Additionally, we evaluated the scores of related gene sets and found that PQQ effectively reversed the age‐related increases in the inflammatory response and TNF signaling pathways in the SnC cluster (Figure S6E–F). In summary, our scRNA‐seq analysis showed a strong association between SnCs and the oxidative stress response. Furthermore, PQQ has the potential to effectively induce senolysis and mitigate the pathological effects caused by SnCs.

**FIGURE 7:**
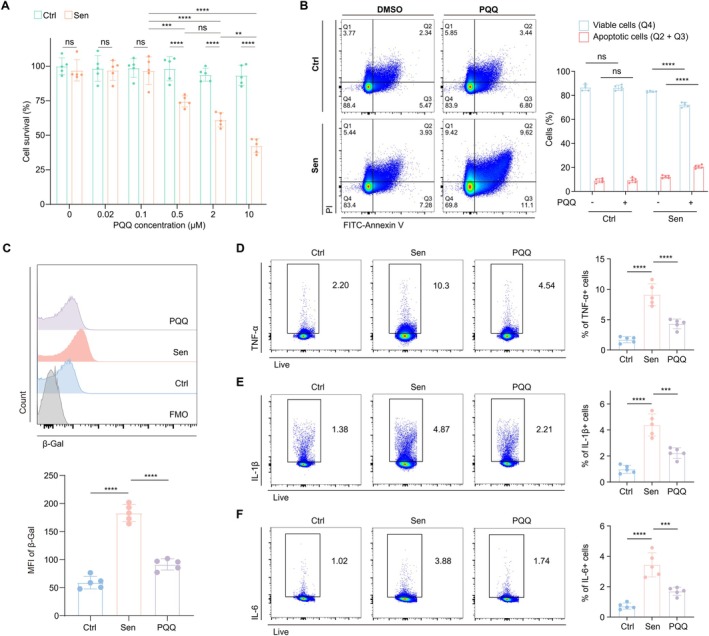
The effects of PQQ in vitro. (A) Bar plot showing the CCK‐8 assay on control and SnCs of RAW 264.7 cell line upon treatment of different concentrations of PQQ (*n* = 5/group). (B) The FCM histograms (left) and column charts (right) show the Annexin V/PI apoptotic assay on control and SnCs of RAW 264.7 cell line upon treatment with PQQ (*n* = 5/group). (C) The FCM histograms (up) and column charts (down) show the MFI of β‐Gal in the RAW 264.7 cell line among the three groups (*n* = 5/group). (D–F) The FCM histograms (left) and column charts (right) show the levels of TNF‐α (D), IL‐1β (E), and IL‐6 (F) in the RAW 264.7 cell line among the three groups (*n* = 5/group). Data are shown as mean ± SD. P values were analyzed using one‐way ANOVA (A‐F); ns, non‐significant, *p < 0.05, **p < 0.01, ***p < 0.001, ****p < 0.0001.

Considering that scRNA‐seq data have confirmed the ability of the antioxidant PQQ to effectively clear SnCs, we next investigated the effects and mechanisms of PQQ on SnCs clearance in vitro. The RAW 264.7 macrophage cell line was selected for in vitro cellular senescence induction and PQQ treatment (see Materials and Methods for details). CCK‐8 assays demonstrated that PQQ, at concentrations up to 10 μM, did not significantly affect the activity of control cells. However, for SnCs, PQQ exhibited cytotoxicity starting at 500 nM, with the effect becoming more pronounced as the concentration increased (Figure 7A). Based on these findings, we selected 500 nM as the treatment concentration to further examine the senolytic and senomorphic effects of PQQ. Next, we performed apoptosis analysis based on Annexin V and propidium iodide (PI) staining, which yielded results consistent with the cell viability assays (Figure 7B), confirming the selective pro‐apoptotic properties of PQQ in SnCs. Furthermore, PQQ treatment significantly reduced the senescence burden, as evidenced by a notable decrease in β‐Gal+ cells (Figure 7C). We next assessed the levels of inflammatory cytokines in aging‐induced cells treated with PQQ. The results showed that PQQ treatment reduced the expression of SASP cytokines, including TNF‐α, IL‐1β, and IL‐6 (Figure 7D–F), thus mitigating the inflammatory phenotype of SnCs. Together, these findings validate the senolytic and senomorphic properties of PQQ.

## 3 Discussion

Aging is a universal and irreversible biological process characterized by the gradual decline in the normal physiological functions of various systems, including the immune system. Oxidative stress can accelerate aging by driving ROS accumulation, which causes cellular damage and promotes inflammation, leading to tissue dysfunction and age‐related diseases (J. Yang et al. 2024). However, the specific effects of oxidative stress on HIS aging and the potential benefits of antioxidants remain unclear. This study employed scRNA‐seq analysis and validation experiments to provide a comprehensive examination of the aging‐related alterations within the HIS and introduces PQQ as a promising therapeutic agent for reversing these effects. Here, we summarized the primary findings as follows: (1) Aging‐related processes in the HIS were altered following aging, with a notable increase in oxidative stress; (2) Long‐term antioxidant PQQ treatment effectively improved physical parameters and reduced circulating levels of SASP factors in the AM group; (3) PQQ exhibited antioxidant, anti‐aging, and anti‐inflammatory effects on various immune cells of HIS; (4) PQQ reduced oxidative stress‐induced apoptosis and senescence processes in specific BC subsets, thereby promoting immune homeostasis; (5) PQQ restored age‐induced alterations in the differentiation and proliferation of HSCs; (6) The identification of SnCs via scRNA‐seq suggested that oxidative stress was closely linked to cellular senescence, and PQQ could exert senolytic effects while suppressing their inflammatory responses.

Aging is primarily characterized by increased oxidative stress, a key driver of cellular senescence and immune dysfunction (Liguori et al. 2018; Terao et al. 2022). Oxidative stress accelerates aging by promoting mitochondrial dysfunction, disrupting energy metabolism, and impairing DNA repair (Zhang, Mack, et al. 2020), contributing to the development of age‐related diseases such as neurodegenerative disorders, cardiovascular diseases, and cancer (Guo et al. 2022). However, its role in HIS aging remains underexplored. In our study, we observed significant increases in SASP, oxidative stress, and inflammatory responses in immune cells of AM, along with elevated expression of interferon family members and other inflammatory genes like Il1b and Ccl4. IL‐1β could promote cellular senescence and tissue inflammation by activating inflammatory pathways (Mantovani et al. 2019), and CCL4 plays a key role in amplifying inflammation by recruiting and activating immune cells, thus maintaining chronic inflammation during aging (Chang et al. 2024). Additionally, the downregulation of immune‐regulatory genes (Tgfb1, Tgfbr2, Foxo1, and Txnip) suggests damage to immune balance with aging. Notably, several studies have reported that elevated Tgfb1 is a key SASP factor contributing to cellular senescence by paracrine mechanism, while others have observed a decline in Tgfb1 during aging (Chen et al. 2021; Haga et al. 2024; Matsuda et al. 2023; D. Sun et al. 2014; Tazawa et al. 2021; Tominaga and Suzuki 2019). Our findings, at the RNA level, reveal a downregulation of Tgf‐related genes in the aging HIS. This aligns with previous research suggesting that decreased Tgf‐β signaling contributes to immune response disorder and HSC regeneration inhibition (Sanjabi et al. 2017; Tominaga and Suzuki 2019). However, the exact role of Tgfb1 in aging remains debated, and further studies are needed to clarify its functions and potential therapeutic implications across different cell types and environmental contexts. Furthermore, the downregulation of the stemness marker Sox4, a critical marker for stem cell and ProBC differentiation, highlights the impairment of cell stemness and early‐stage cell differentiation during aging (Braccioli et al. 2018; Sun et al. 2013). Overall, these findings underscore the significant role of aging in promoting abnormal inflammatory responses and immune dysregulation.

Previous research has shown that ROS impairs T cell function by affecting proliferation, differentiation, and immune responses (Yarosz and Chang 2018). Regulating oxidative stress can preserve immune cell function, enhance immune surveillance, and prevent senescence (Martínez de Toda et al. 2021). PQQ, an amino acid‐based potent antioxidant, has demonstrated the ability to scavenge free radicals and reduce oxidative stress, making it a promising agent in mitigating age‐related immune dysfunction (Jonscher et al. 2021). In a clinical study conducted by Rucker and Harris et al., oral supplementation with PQQ enhances the body's antioxidant capacity, reduce inflammatory factors levels, and improve mitochondrial function (Harris et al. 2013). Building on human‐to‐mouse dosage conversion, we selected the human‐equivalent dosage for long‐term supplementation and found it significantly improved the physiological parameters of AM and exerted antioxidant, anti‐aging and immunomodulatory effects on a variety of immune cells. These results were consistent with previous findings that antioxidants can extend lifespan and enhance physiological functions (Tuo et al. 2023; Yang et al. 2023). Moreover, by employing scRNA‐seq analysis and machine learning, we found that SnCs of aged mice could be cleared effectively by PQQ and found that increased oxidative stress was associated with the accumulation of SnCs. We also found a significantly higher proportion of SnCs in myeloid cells and granulocytes. This finding is consistent with the higher levels of p16, p53 and SASP in myeloid cells compared to other immune cell types (Farr et al. 2016). And this is further supported by a recent report that physiologically senescent monocytes/macrophages propagate senescence processes to multiple tissue and drive age‐related dysfunction via extracellular vesicles (Hou et al. 2024). Furthermore, oxidative stress drives the accumulation of ROS, which accelerates the generation of SnCs and the production of the SASP, leading to heightened inflammatory responses, particularly in myeloid cells (Schieber and Chandel 2014; Trombetti et al. 2021). Thus, the senolytic effects of antioxidant PQQ on SnCs further elucidate the key role of oxidative stress on SnCs. The senolytic activity of PQQ stems from its selective pro‐apoptotic effects on SnCs, resembling the mechanisms observed in traditional senescent cell clearance agents (Di Micco et al. 2021). We also found that PQQ inhibited inflammatory cytokine production and related pathways, as well as modulating several normal physiological and metabolic processes in SnCs. Therefore, compared to canonical single‐function senolytics, PQQ can also directly inhibit upstream signaling oxidative stress and therefore the release of SASPs, thereby further expanding our understanding of the mechanisms underlying the anti‐aging properties of PQQ (Hao et al. 2020). Our data demonstrate that elevated oxidative stress is a key mechanism driving HIS aging and SnCs accumulation and pathogenicity and highlight the senolytic and senomorphic effects of PQQ.

bcs play a crucial role in the adaptive immune system, contributing to the production of antibodies and the establishment of memory immune response (Akkaya et al. 2020; Sabatino Jr. et al. 2019). However, previous studies have shown that aging impairs bc function, leading to a reduction in BCs diversity, increased differentiation and inflammation, and a decreased ability to respond to new antigens (de Mol et al. 2021; Xie et al. 2021). In BCs, oxidative stress can disrupt antibody production and maturation, which impairs the body's ability to respond to infections and create long‐lasting immunity (Zhang et al. 2019). In the current study, we found that the antioxidant PQQ reversed the aging‐associated changes in BCs. Specifically, PQQ influenced key pathways such as BCs proliferation and BC receptor signaling pathway. In addition to its anti‐aging effect, PQQ also supported function‐associated phenotypes in BCs by modulating pathways like IL‐7 signaling, which are critical for BCs survival. Therefore, our findings indicate that PQQ exerts anti‐aging, anti‐inflammatory, and antioxidant effects in BCs, promoting immune regeneration and restoring BCs function and homeostasis. Furthermore, our study revealed that aging‐induced oxidative stress promoted apoptosis in bc progenitors, specifically in PreBC and ProBC, thereby compromising the immune response. PreBC and ProBC, as early stages in BCs development, are highly dependent on IL‐7 signaling for survival and differentiation. The IL‐7 receptor IL‐7R on these developing subsets can bind cytokine IL‐7, thereby activating key pathways that promote BCs survival, proliferation, and differentiation (Corfe and Paige 2012; Kaiser et al. 2023). Therefore, our use of IL‐7R as a marker for PreBC and ProBC allowed us to specifically track the effects of PQQ on these critical BCs subpopulations. Our findings indicated that PQQ supplementation effectively restored IL‐7R levels in the BCs of aged mice, thereby promoting BC regeneration. Furthermore, we identified and validated that the apoptosis‐related factor ASPP1 was associated with BCs aging and may serve as a target for PQQ intervention. ASPP1 acts as a regulator of p53‐mediated apoptosis (Schittenhelm et al. 2024), and its upregulation in aged BCs suggests an increased susceptibility to apoptosis due to heightened oxidative stress. Notably, we observed that PQQ downregulates ASPP1 levels in both overall BCs and IL‐7R+ BCs, underscoring its potential to inhibit BCs apoptosis, preserve BCs subpopulations, and maintain immune homeostasis. In summary, by reversing oxidative stress‐induced alterations in BCs subpopulations, PQQ contributes to a balanced immune response, ensuring BCs hemostasis and effective activation against new antigens, which is critical for the immune system's adaptability.

HSCs are multipotent stem cells responsible for the continuous regeneration of blood and immune cells (Hawley et al. 2006). As the body ages, HSCs experience a decline in their self‐renewal capacity and show a shift toward myeloid‐biased differentiation, leading to reduced production of lymphocytes (Mejia‐Ramirez and Florian 2020). Our recent article revealed significant transcriptional changes in HSCs through scRNA‐seq, identifying CD62L+ HSCs as a potential player in HIS aging. In the current study, we demonstrated that the aging induced HSCs to obtain phenotypes related to inflammatory responses and oxidative stress. Previous studies have underscored that the reduction in oxidative stress not only helps reverse HSC aging but also exhibits therapeutic effects for various age‐related diseases, such as anemia and myeloid malignancies (Al Balushi et al. 2019; Trombetti et al. 2021). Therefore, our study investigated the impact of long‐term PQQ administration on HSCs. We found that PQQ inhibited the overactivated inflammation and increased the levels of adiponectin receptors (Adipor1, Adipor2). It has been reported that adiponectin receptor signaling attenuates the inflammatory response mediated by myeloid cells, consequently mitigating the loss of HSCs self‐renewal potential induced by inflammation (Meacham et al. 2022). Therefore, PQQ can indirectly promote the physiological function of HSC by regulating the immune cells' activation. We also found that PQQ directly influenced aged HSCs by reversing age‐related changes, including apoptosis and oxidative stress. In addition, PQQ supplementation also reversed G1/S phase arrest, promoting the cell cycle and proliferative potential of HSCs. Aging is associated with an arrest in the G1/S phase of the cell cycle, leading to a decrease in HSC proliferation (Mejia‐Ramirez and Florian 2020). Based on the positive effects of PQQ on the cell cycle dynamics of HSCs, we further identified the potential target Yy1, which was decreased in aged HSC and increased after PQQ treatment. Yy1, in particular, is a transcriptional regulator that plays a crucial role in maintaining the long‐term repopulating activity of HSCs (Lu et al. 2018). We validated that PQQ promoted Yy1 expression, therefore contributing to the rejuvenation of aged HSCs. Moreover, we observed that PQQ significantly restored the reduced expression of two key markers, CD62L and CD34, which are essential for preserving the homing ability, self‐renewal, and differentiation capacity of HSCs (Guo et al. 2003; Sottoriva and Pajcini 2021). Overall, our study indicates the aging‐related alterations in HSCs of HIS and highlights that PQQ can reverse age‐related changes and restore the expression of key molecules to support their normal physiological functions and immune homeostasis.

In conclusion, this study provides a detailed characterization of the aging‐related changes in the HIS and identifies oxidative stress as a key factor in HIS aging. Furthermore, we explore the antioxidant PQQ as a promising therapeutic agent for counteracting the effects of aging by mitigating oxidative stress and exhibiting senolytic and senomorphic properties. This highlights its potential in combating age‐related immune dysfunction and restoring normal physiological functions of immune cells. This study not only advances our understanding of the aging immune system but also opens new avenues for the development of targeted interventions to promote healthy aging and enhance longevity.

## 4 Materials and Methods

### 4.1 Mice and PQQ Treatment

2‐3‐week‐old (young mice, YM), 15‐17‐month‐old, and 19‐21‐month‐old (aged mice, AM) C57BL/6J mice were purchased from SPF Biotechnology Co. Ltd. (Beijing, China). Animal experiments were approved by the Institutional Animal Care and Use Committee at Zhongshan Ophthalmic Center, Sun Yat‐sen University. All mice were housed in a 12‐h light/dark cycle. The 15‐17‐month‐old mice were divided into the following two groups: (1) Aged group with 15‐17‐month‐old mice received standard water and food ad libitum. (2) PQQ‐treated group with 15‐17‐month‐old mice received water and tailor‐made food ad libitum. PQQ (#S3578, Selleck, Houston, TX, USA) was mixed with the regular chow with a concentration of 18 mg/kg, which was an approximate average of 3.6 mg/kg·d for each mouse, a dosage similar to that used in clinical studies for humans according to the FDA guidelines (Alert 2005; Harris et al. 2013). The administration of PQQ lasted for 4 months before euthanasia.

### 4.2 Cells Isolation and Preparation

For scRNA‐seq, spleen and bone marrow cells from four YM mice and four AM mice were pooled into a single sample to ensure adequate cell numbers for sequencing. Bone marrow cells were isolated by dissection of the femurs and tibias. The surrounding muscles and periosteum were carefully removed. Bone marrow was flushed from the femurs and tibias using RPMI‐1640 cell culture media (Gibco) supplemented with 1% penicillin/streptomycin (Life Technologies) and 10% fetal bovine serum (Gemini Bio Products, Sacramento, CA). The resulting cell suspension was filtered through a 40 μm cell strainer (Corning) to yield a single‐cell suspension and centrifuged at 1200 rpm for 5 min. For spleen cell isolation, the spleens were detached from the abdominal cavity of mice and placed on ice in the RPMI‐1640 media. The spleens were then filtered through a cell strainer to obtain a single‐cell suspension. Red blood cells were lysed using a red blood cell lysis buffer composed of 144 mM NH_4_Cl and 17 mM Tris (pH 7.6).

### 4.3 Cell Culture and Treatment

The RAW 264.7 macrophage cell line, obtained from Zhong Qiao Xin Zhou Biotechnology (Shanghai, China), was cultured in DMEM (Gibco) supplemented with 10% fetal bovine serum and 1% penicillin/streptomycin. Cells were maintained at 37°C with 5% CO_2_ under normal culture conditions. To induce cellular senescence, 200 μM H_2_O_2_ (#7722‐84‐1, Sigma‐Aldrich, St. Louis, MO, USA) was added to the complete medium for 4 h daily over four consecutive days. Afterward, cells were transferred to fresh complete medium and cultured for an additional 2 days before harvesting. For PQQ treatment, cells were exposed to 500 nM of PQQ for 24 h.

### 4.4 Cell Viability Assay

Senescent or control cells (5 × 10^4^ /well) were plated in a 96‐well plate and treated with different concentrations of PQQ (0–10 μM). After 24 h of incubation at 37°C with 5% CO_2_ in a humidified chamber, cell viability was assessed using a Cell Counting Kit‐8, following the manufacturer's guidelines.

### 4.5 Enzyme‐Linked Immunosorbent Assay (ELISA)

Mouse serum levels of TNF‐α and CCL4 were measured using the mouse TNF‐α ELISA Kit (#88–7324‐88, Invitrogen) and the mouse CCL4 ELISA Kit (#E‐UNEL‐M0132, Elabscience Biotechnology Co. Ltd.), following the manufacturer's instructions.

### 4.6 Flow Cytometry (FCM) Analysis

The BM and SP cells were assigned for FCM analysis. A live/dead cell dye (#L34968, Invitrogen; or #423105, BioLegend, San Diego, CA, USA) was used to exclude dead cells. Cells were then stained with the following surface markers antibodies: CD45 APC (#103112), CD45 BV510 (#103138), CD3 PE/CF594 (#100246), CD19 BV650 (#115541), IL‐7R (#135014), Lineage AF700 (#133313), SCA‐1 APC/Cy7 (#108125), CD117 PE/Cy7 (#105813), CD62L FITC (#104405), CD34 BV421 (#152207), CD11C PerCP/Cy5.5 (#117328), LY6C APC (#128016), CD11B BV421 (#101235), LY6G BV605 (#127639), and F4/80 PE/Cy7 (#123114). For intracellular molecule staining, the cells were stimulated with 5 ng/mL phorbol myristate acetate, 500 ng/mL ionomycin, and 1 mg/mL brefeldin A (Sigma) at 37°C and 5% CO2 for 5 h, followed by fixation for 45 min and permeabilization for 30 min. Then, cells were stained with the following antibodies: TNF‐α BV421 (#506327, BioLegend), IL‐1β (#25–7114‐80, Invitrogen, Carlsbad, CA, USA), and IL‐6 PE (#504504, BioLegend). For ASPP1 and Yy1 staining, cells were incubated with the ASPP1 primary antibody (#bs‐1282R, BIOSS) and Yy1 primary antibody (#22156‐1‐AP, Proteintech) overnight, and then stained with one of the following second antibodies for 4 h: AF488‐labeled antibody (#4412S) or AF647‐labeled antibody (#4414S). The antibody dilutions used in this study were mainly determined based on the instructions. Finally, the cells were stored at 4°C overnight in preparation for FCM analysis. For the β‐galactosidase (β‐Gal) staining, cells were stained with the senescence assay kit (#ab228562, Abcam) and harvested for FCM analysis according to the manufacturer's instructions. For Annexin V and Propidium iodide (PI) staining, cells were stained with the apoptosis assay kit (#556547, BD Biosciences, San Jose, CA, USA) according to the manufacturer's instructions. After analysis using a flow cytometer (BD LSRFortessa, USA), the FCS data were analyzed using FlowJo software (version 10.0, Tree Star, Ashland, OR, USA).

### 4.7 RNA Isolation and Real‐Time Quantitative PCR

Total RNA of the whole retinas was extracted by Trizol, and then the level was measured with a NanoDrop spectrophotometer. Next, cDNAs were synthesized using the PrimeScript RT Master Mix. Real‐time quantitative PCR was performed using SYBR Premix Ex TaqTM II (TaKaRa Bio Inc.). The primer sequences were provided in Table S1. The relative mRNA levels of indicated genes were determined using the 2–ΔΔCt method based on the control level of β‐actin mRNA.

### 4.8 scRNA‐Seq

#### 4.8.1 scRNA‐Seq Data Processing

Single‐cell suspensions of bone marrow and spleen cells were subjected to the Chromium Single Cell 5' Library (10× Genomics, Genomics chromium platform Illumina NovaSeq 6000) Chip Kit and Gel Bead Kit (10× Genomics) to generate scRNA libraries following the manufacturer's protocol. FastQC software was used to check the primary quality of the library and preliminarily process the sequencing data. CellRanger Software (Version 4.0; 10× Genomics), where multiple sequences were separated and barcoded using a counting pipeline in the CellRanger software for the initial processing of sequencing data. The Seurat package (version 4.3.0) was used to perform dimensionality reduction and clustering analysis using default parameters. Quality control was performed to exclude cells with fewer than 200 detected genes and those with a percentage of mitochondrial genes greater than 15%. A total of 32,894 cells (YM, 7385 cells; AM, 8736 cells; PQQ, 16,773 cells) were used for subsequent analysis. ‘NormalizeData’ and ‘ScaleData’ functions were used to logarithmically normalize and scale the data respectively. After ‘Harmony’ was used for batch effect correction, we dimensionalized the data by the ‘RunPCA’ and used ‘FindVariableFeatures’ to calculate high variability genes. Cell clustering was then performed by ‘FindClusters’ and ‘FindNeighbors’. ‘RunTSNE’ was used to show cell clustering results in t‐SNE plots, and ‘FindAllMarker’ was used to identify preferentially expressed genes in clusters.

#### 4.8.2 Differentially Expressed Genes (DEGs) Analysis

Differentially expressed genes (DEGs) between YM and AM groups, or AM and PQQ groups, were identified using the ‘FindMarkers’ function from the Seurat package, employing criteria of *p* < 0.05 and |Log2 fold‐change| > 0.25. Prior to the DEGs analysis, we excluded any cell types that were either missing or had fewer than three cells in the comparison groups.

#### 4.8.3 Gene Ontology (GO) Analysis

Gene Ontology (GO) biological processes and pathway analyses were conducted using the Metascape tool to visualize the functional patterns of the DEGs. Among the top 50 enriched GO terms across various cell subtypes, 10 terms related to inflammation or aging were selected for visualization and analysis using the ggplot2 R package.

#### 4.8.4 Scoring of Biological Processes

Using “AddModuleScore” function in the Seurat R package (version 4.3.0), the individual cells were scored for their expression of gene signatures that represented certain biological functions. The genes related to oxidative stress response were obtained from Wikipathways Pathways (WP408). The inflammatory response score was calculated based on the genes in the GO term “inflammatory response” (GO: 0006954). Genes related to cell division were obtained from the GO term “cell division” (GO: 0051301). The genes related to SASP were obtained from Reactome Pathways (R‐HSA‐2559582), and the genes related to TNF signaling pathway were obtained from Wikipathways Pathways (WP231). The genes used for biological process scoring are detailed in Tables S2–S6.

#### 4.8.5 Cell Cycle Evaluation

Forty‐three G1/S genes and 54 G2/M genes, previously identified as core gene sets, were used to perform cell cycle analysis using the “CellCycleScoring” function in the Seurat R package.

#### 4.8.6 Statistical Analysis

The two‐tailed unpaired Student's t‐test was employed to compare variables between two groups. For comparisons involving three or more groups, one‐way ANOVA or two‐way ANOVA with Bonferroni post‐tests were utilized to assess differences in one variable or multiple variables, respectively. The results were represented as mean ± SD. The statistical analysis was performed using GraphPad Prism software (version 8.0.2; GraphPad Software Inc.). The data analysis pipeline for scRNA‐seq followed protocols outlined on the official 10X Genomics and Seurat websites. For multiple group comparisons of gene levels or functional scores in scRNA‐seq analysis, we applied the default Holm correction method in the one‐way ANOVA, utilizing the “compare_means” function from the “ggpubr” R package with default parameters. P‐values for GO biological processes and pathway terms were calculated using the hypergeometric test with default parameters in the Metascape web tool. P‐values greater than 0.05 were considered statistically insignificant and denoted as NS. Significance levels were indicated as follows: *p < 0.05, **p < 0.01, ***p < 0.001, ****p < 0.0001. Experimental findings were consistently reproduced in two independent experiments, with the number of samples used for statistical analysis detailed in the figure legends.

## Author Contributions

**Zhuping Xu:** conceptualization; formal analysis; supervision; funding acquisition; investigation; visualization; methodology; project administration. **Wenru Su:** conceptualization; funding acquisition; data curation; supervision; visualization; methodology; project administration. **Xiuxing Liu:** conceptualization; formal analysis; validation; investigation; visualization; methodology; writing – original draft. **Chun Zhang:** conceptualization; data curation; validation; investigation; visualization; methodology; writing – original draft. **Jianjie Lv:** data curation; investigation; methodology. **Yidan Liu:** data curation; formal analysis; writing – original draft. **Chenyang Gu:** validation; investigation. **Yuehan Gao:** writing – review and editing. **Wen Ding:** writing – review and editing. **Hui Chen:** methodology. **Nanwei Xu:** investigation. **Hongbin Yin:** investigation.

## Ethics Statement

All experiments were performed in compliance with the ARVO Animal Statement for the Use of Animals in Ophthalmic and Vision Research.

## Conflicts of Interest

The authors declare no conflicts of interest.

## Supporting information


Appendix S1.

Appendix S2.

## Data Availability

The scRNA‐seq data is deposited in the Genome Sequence Archive in BIG Data Center, Beijing Institute of Genomics (BIG, https://bigd.big.ac.cn/gsa/), Chinese Academy of Sciences. The data of YM and AM were obtained from the GSA Accession No. CRA012362. The data of PQQ‐treated mice was deposited under the GSA Accession No. CRA018973.
