# Supplementary material for: Pyrroloquinoline Quinone Reprograms the Single‐Cell Landscape of Immune Aging in Hematopoietic Immune System
Source: Aging Cell. 2025 Apr 7;24(7):e70050. doi: 10.1111/acel.70050 (PMC12266787; doi:10.1111/acel.70050)
Supplement: Supplementary file 1 — Appendix S1. [file ACEL-24-e70050-s002.pdf]

Figure S1. The scRNA-seq analysis on HIS cells of YM and AM group.
A-B. Violin plots showing the score of inflammatory response (**A**) and cell division (**B**) between two groups.
Data are shown as mean ± SD. P values were analyzed using Student's t-test (A-B); ****P< 0.0001.

Figure S2. The effects of PQQ on physiological index and HIS cells.
A-D. Violin plots showing the score of SASP (**A**) and oxidative stress response (**B**), inflammatory response (**C**), and cell division (**D**) between the two groups.
E. Line graph showing the ratio of cell subpopulations among the three groups.
F. The FCM histograms showing the gating strategies for BC and the BC-derived molecules.
G. The FCM histograms showing the gating strategies for LSK and the LSK-derived molecules.
Data are shown as mean ± SD. P values were analyzed using Student's t-test (A-D); **P< 0.01, ****P< 0.0001.

Figure S3. PQQ reverses aging-related gene expression changes in HIS cells.
A-D. Representative GO biological processes and pathways enriched in upregulated aging-DEGs (**A**), downregulated PQQ-DEGs (**B**), downregulated aging-DEGs (**C**), and upregulated PQQ-DEGs (**D**) in HIS cells.
E. Bar chart showing the mRNA levels of SASP-related genes.
F. Volcano plot showing the up- or down-regulated rescue-DEGs in BASO subset.

Figure S4. The effects of PQQ on BC.
A-C. Violin plots showing the score of SASP (**A**), inflammatory response (**B**), and cell division (**C**) among the three groups.
D. Heatmap showing scaled expression of discriminative gene sets for each BC subset.
E. Bar chart showing the ratio of rescue-DEGs to aging-DEGs in each BC subset.
F. Violin plots showing the levels of Il7r among BC subsets.
G. The FCM histograms showing the percentage of IL-7R and ASPP1 in SP BC among the three groups.
H. The column charts showing the percentage of SP IL-7R+ BC (left), SP ASPP1+ BC (mid), and ASPP1 in SP IL-7R+ BC (right) among the three groups (n = 5/group).
Data are shown as mean ± SD. P values were analyzed using one-way ANOVA (A-C, H); *P< 0.05, **P< 0.01, ****P< 0.0001.

Figure S5. The effects of PQQ on HSC.
A-B. Violin plots showing the score of SASP (**A**) and inflammatory response (**B**) among the HSC of three groups.
C. The scatter plot showing the lymphoid or myeloid differentiation scores of hematopoietic stem cells, lymphocytes, and myeloid cells.
D. The scatter plot showing the classification of HSC into the three phases (G1, S, and G2M) based on the cell-cycle score of G1/S and G2/M gene sets.
E-H. The FCM histograms (left) and column charts (right) showing the

percentage of SP Yy1+ LSK (**E**), SP CD34+ LSK (**F**), and MFI of CD62L in BM LSK (**G**) and SP LSK (**H**) among the three groups (n = 5/group).
Data are shown as mean ± SD. P values were analyzed using one-way ANOVA (A-B, E-H); *P<0.05, **P< 0.01, ***P< 0.001, ****P< 0.0001.

Figure S6. The effects of PQQ on SnC.
A. The pie chart showing the proportion of SnC in HIS cells.
B. The FCM histograms showing the gating strategies for β-Gal expression in immune cell, BC, macrophage, DC, and neutrophil.
C. Representative GO biological processes and pathways enriched in upregulated PQQ-DEGs in SnC.
D-F. Violin plots showing the score of oxidative stress response (**D**), inflammatory response (**E**), and TNF signaling pathway (**F**) among the three groups.
Data are shown as mean ± SD. P values were analyzed using one-way ANOVA (D-F); *P<0.05, **P< 0.01, ****P< 0.0001.

Figure S1

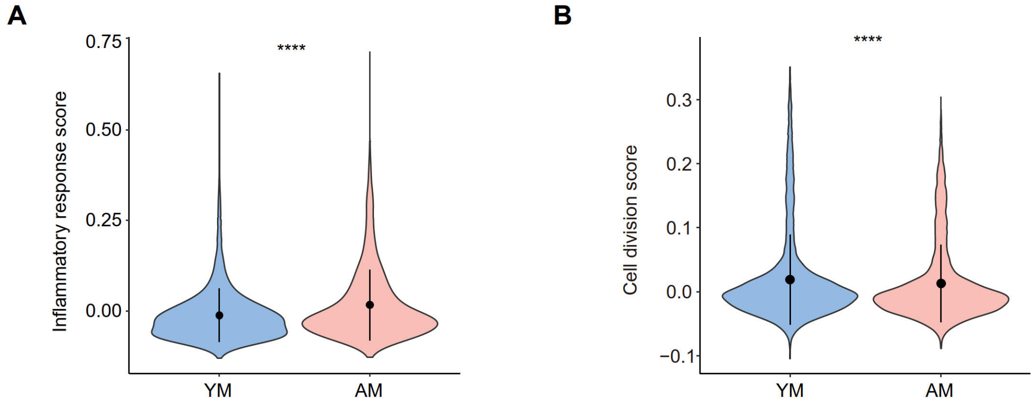
A
Inflammatory response score
0.75
0.50
0.25
0.00
****
YM
AM
B
Cell division score
0.3
0.2
0.1
0.0
−0.1
****
YM
AM

Figure S2

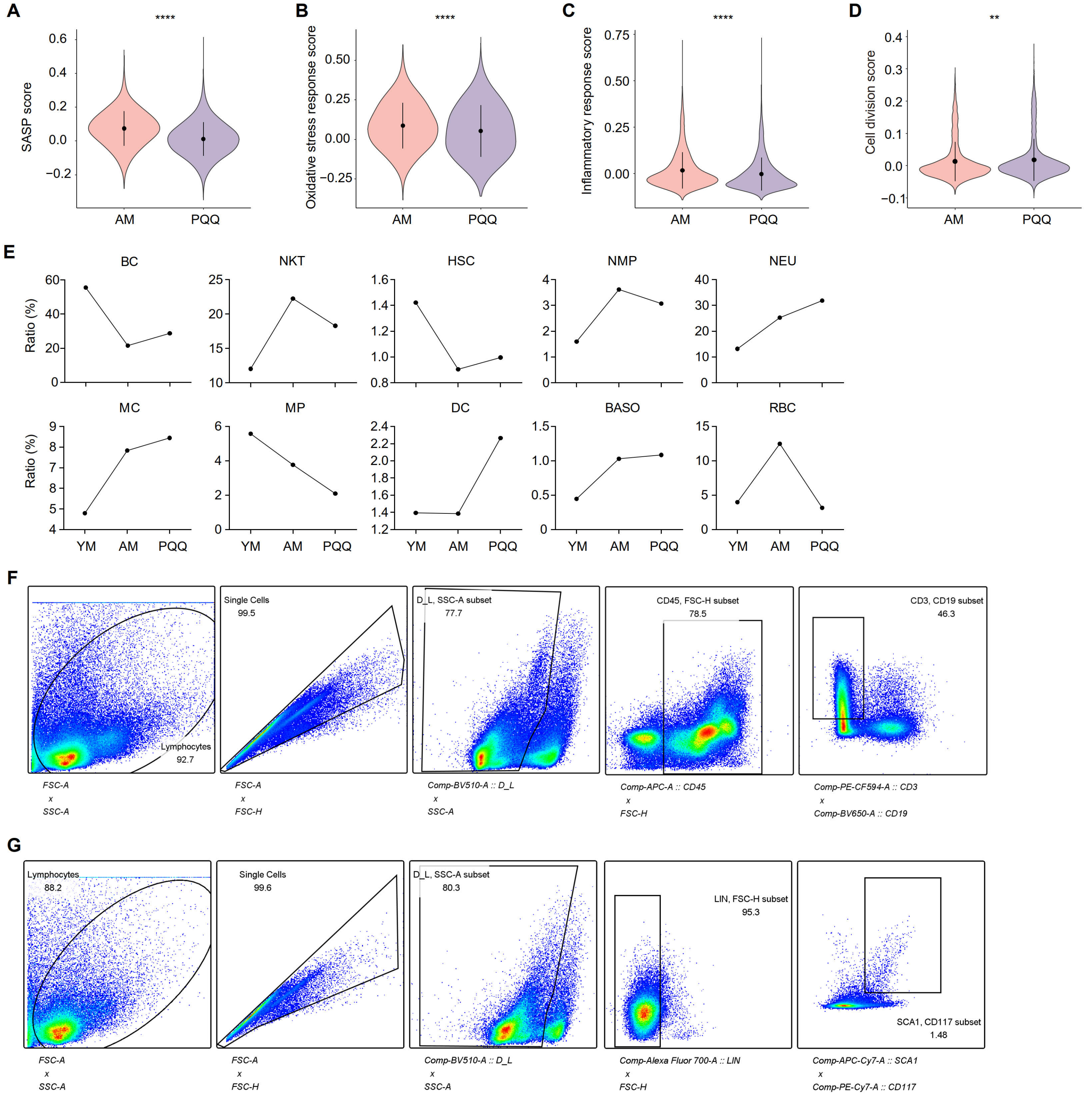
A
****
SASP score
0.6
0.4
0.2
0.0
−0.2
AM
PQQ
B
****
Oxidative stress response score
0.50
0.25
0.00
−0.25
C
****
Inflammatory response score
0.75
D
**
Cell division score
0.4
0.3
0.1
−0.1
E
BC
NKT
HSC
NMP
NEU
MC
MP
DC
BASO
RBC
Ratio (%)
YM
F
Single Cells
99.5
D_L, SSC-A subset
77.7
CD45, FSC-H subset
78.5
CD3, CD19 subset
46.3
Lymphocytes
92.7
FSC-A
x
SSC-A
FSC-H
Comp-BV510-A :: D_L
Comp-APC-A :: CD45
Comp-PE-CF594-A :: CD3
Comp-BV650-A :: CD19
G
Lymphocytes
88.2
Single Cells
99.6
D_L, SSC-A subset
80.3
LIN, FSC-H subset
95.3
SCA1, CD117 subset
1.48
Comp-Alexa Fluor 700-A :: LIN
Comp-APC-Cy7-A :: SCA1
Comp-PE-Cy7-A :: CD117

Figure S3

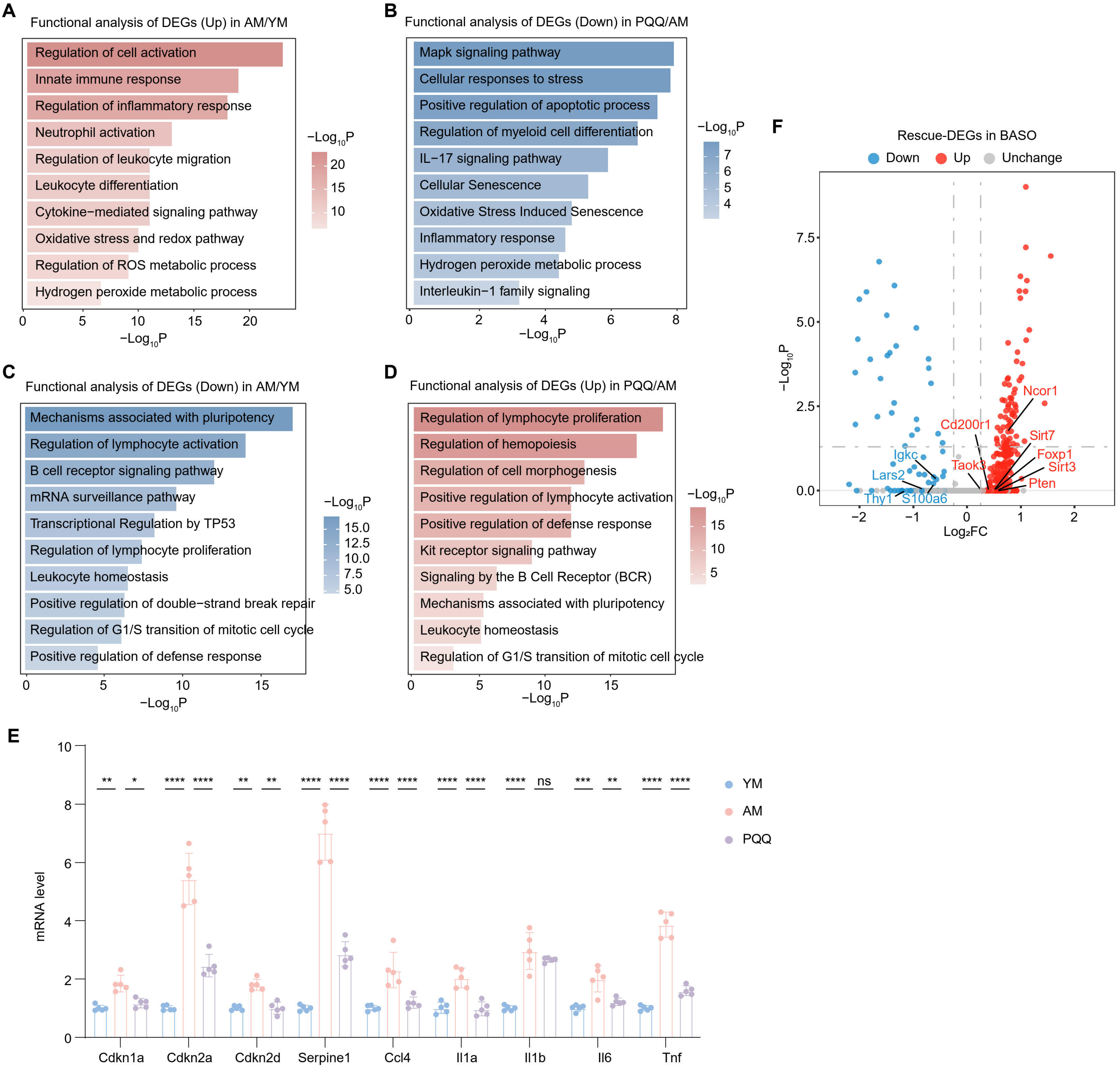
A
Functional analysis of DEGs (Up) in AM/YM
Regulation of cell activation
Innate immune response
Regulation of inflammatory response
Neutrophil activation
Regulation of leukocyte migration
Leukocyte differentiation
Cytokine−mediated signaling pathway
Oxidative stress and redox pathway
Regulation of ROS metabolic process
Hydrogen peroxide metabolic process
−Log10P
B
Functional analysis of DEGs (Down) in PQQ/AM
Mapk signaling pathway
Cellular responses to stress
Positive regulation of apoptotic process
Regulation of myeloid cell differentiation
IL−17 signaling pathway
Cellular Senescence
Oxidative Stress Induced Senescence
Inflammatory response
Hydrogen peroxide metabolic process
Interleukin−1 family signaling
−Log10P
C
Functional analysis of DEGs (Down) in AM/YM
Mechanisms associated with pluripotency
Regulation of lymphocyte activation
B cell receptor signaling pathway
mRNA surveillance pathway
Transcriptional Regulation by TP53
Regulation of lymphocyte proliferation
Leukocyte homeostasis
Positive regulation of double−strand break repair
Regulation of G1/S transition of mitotic cell cycle
Positive regulation of defense response
−Log10P
D
Functional analysis of DEGs (Up) in PQQ/AM
Regulation of lymphocyte proliferation
Regulation of hemopoiesis
Regulation of cell morphogenesis
Positive regulation of lymphocyte activation
Positive regulation of defense response
Kit receptor signaling pathway
Signaling by the B Cell Receptor (BCR)
Mechanisms associated with pluripotency
Leukocyte homeostasis
Regulation of G1/S transition of mitotic cell cycle
−Log10P
E
mRNA level
YM
AM
PQQ
Cdkn1a
Cdkn2a
Cdkn2d
Serpine1
Ccl4
Il1a
Il1b
Il6
Tnf
ns
F
Rescue-DEGs in BASO
Down
Up
Unchange
Ncor1
Cd200r1
Sirt7
Foxp1
Sirt3
Taok3
Pten
Igkc
Lars2
Thy1
S100a6
Log2FC
−Log10P

Figure S4

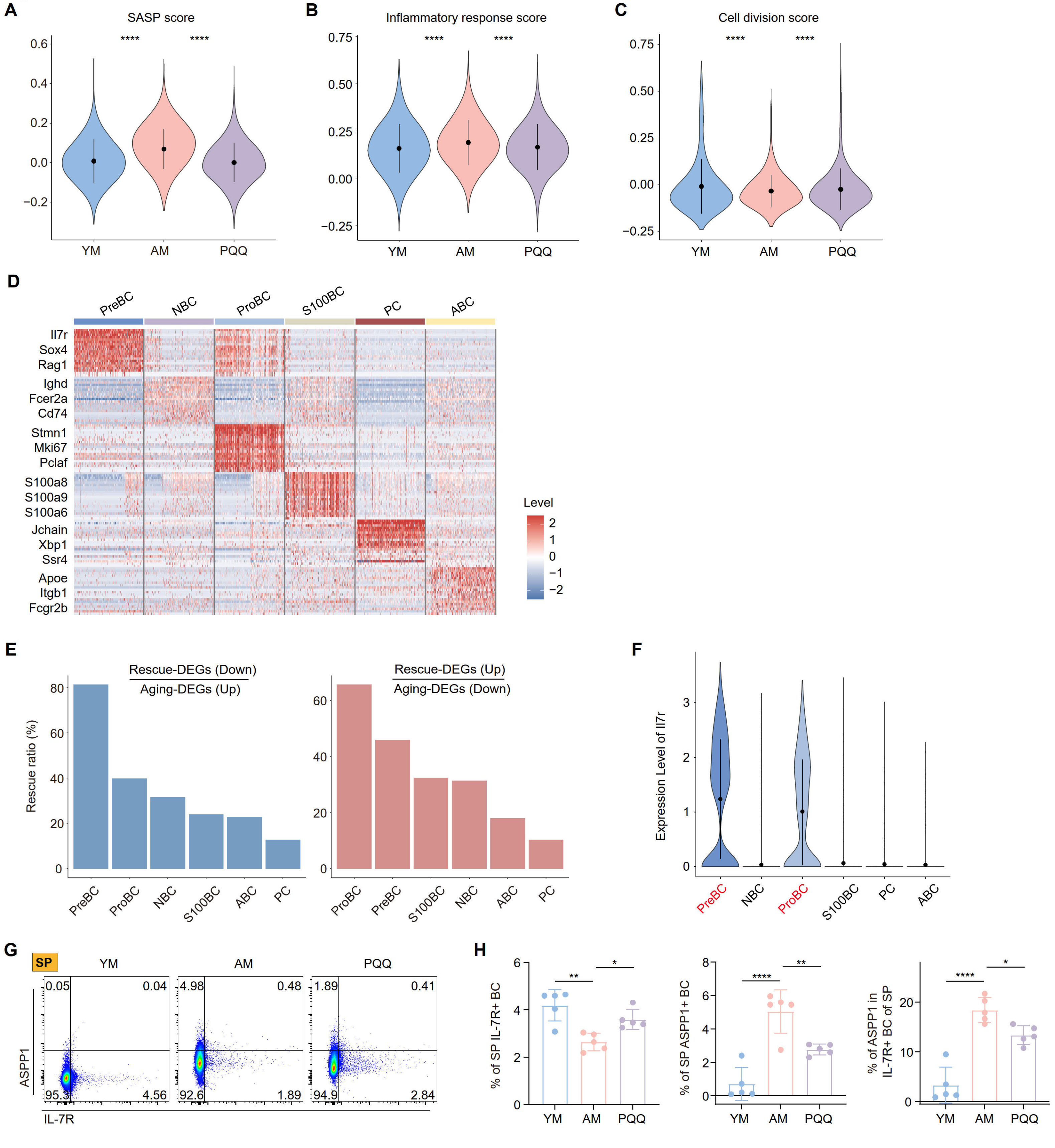
A
SASP score
YM
AM
PQQ
B
Inflammatory response score
C
Cell division score
D
PreBC
NBC
ProBC
S100BC
PC
ABC
Il7r
Sox4
Rag1
Ighd
Fcer2a
Cd74
Stmn1
Mki67
Pclaf
S100a8
S100a9
S100a6
Jchain
Xbp1
Ssr4
Apoe
Itgb1
Fcgr2b
Level
E
Rescue-DEGs (Down)
Aging-DEGs (Up)
Rescue-DEGs (Up)
Aging-DEGs (Down)
Rescue ratio (%)
F
Expression Level of Il7r
G
SP
ASPP1
IL-7R
H
% of SP IL-7R+ BC
% of SP ASPP1+ BC
% of ASPP1 in IL-7R+ BC of SP

Figure S5

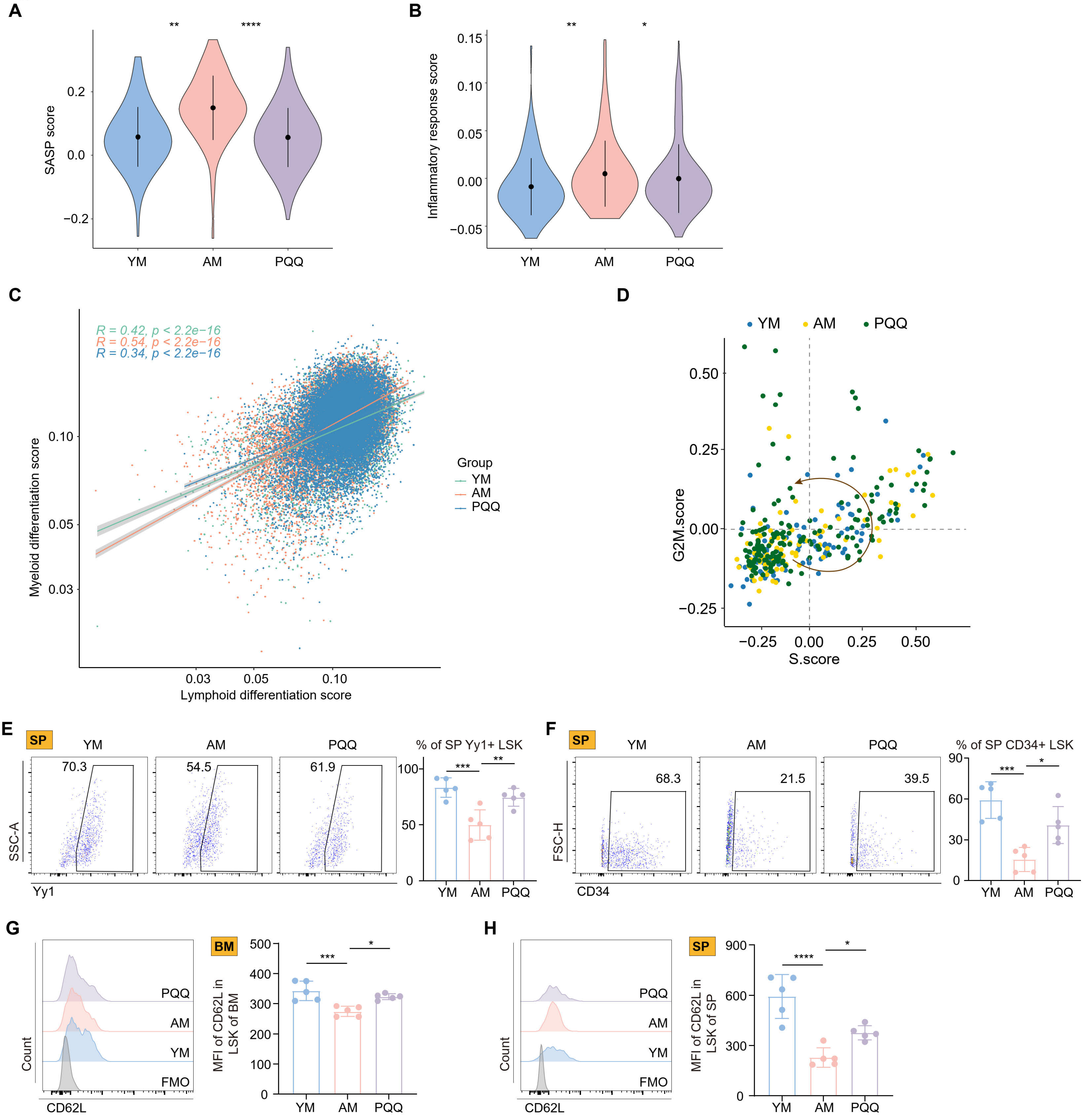
A
SASP score
YM
AM
PQQ
B
Inflammatory response score
C
R = 0.42, p < 2.2e−16
R = 0.54, p < 2.2e−16
R = 0.34, p < 2.2e−16
Myeloid differentiation score
Lymphoid differentiation score
Group
D
G2M.score
S.score
E
SP
% of SP Yy1+ LSK
SSC-A
Yy1
70.3
54.5
61.9
F
% of SP CD34+ LSK
FSC-H
CD34
68.3
21.5
39.5
G
BM
MFI of CD62L in LSK of BM
Count
CD62L
FMO
H
MFI of CD62L in LSK of SP

Figure S6

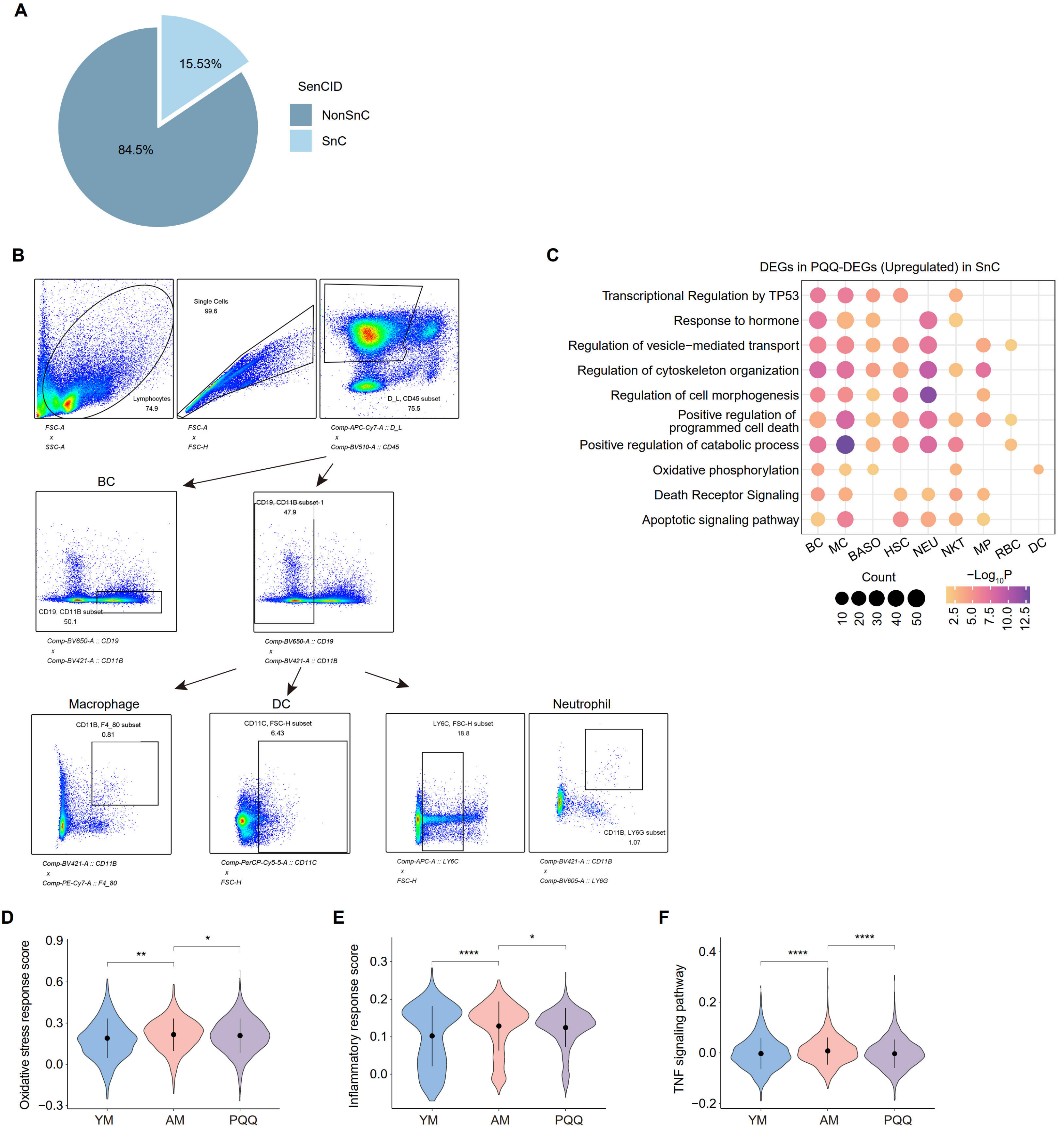
A
15.53%
84.5%
SenCID
NonSnC
SnC
B
Lymphocytes
74.9
FSC-A
x
SSC-A
Single Cells
99.6
FSC-A
x
FSC-H
D_L, CD45 subset
75.5
Comp-APC-Cy7-A :: D_L
x
Comp-BV510-A :: CD45
BC
CD19, CD11B subset
50.1
Comp-BV650-A :: CD19
x
Comp-BV421-A :: CD11B
CD19, CD11B subset-1
47.9
Comp-BV650-A :: CD19
x
Comp-BV421-A :: CD11B
Macrophage
CD11B, F4_80 subset
0.81
Comp-BV421-A :: CD11B
x
Comp-PE-Cy7-A :: F4_80
DC
CD11C, FSC-H subset
6.43
Comp-PerCP-Cy5-5-A :: CD11C
x
FSC-H
Neutrophil
LY6C, FSC-H subset
18.8
Comp-APC-A :: LY6C
x
FSC-H
CD11B, LY6G subset
1.07
Comp-BV421-A :: CD11B
x
Comp-BV605-A :: LY6G
C
DEGs in PQQ-DEGs (Upregulated) in SnC
Transcriptional Regulation by TP53
Response to hormone
Regulation of vesicle−mediated transport
Regulation of cytoskeleton organization
Regulation of cell morphogenesis
Positive regulation of programmed cell death
Positive regulation of catabolic process
Oxidative phosphorylation
Death Receptor Signaling
Apoptotic signaling pathway
BC
MC
BASO
HSC
NEU
NKT
MP
RBC
DC
Count
10
20
30
40
50
−Log10P
2.5
5.0
7.5
10.0
12.5
D
Oxidative stress response score
0.9
0.6
0.3
0.0
−0.3
**
*
YM
AM
PQQ
E
Inflammatory response score
0.3
0.2
0.1
0.0
****
*
YM
AM
PQQ
F
TNF signaling pathway
0.4
0.2
0.0
−0.2
****
****
YM
AM
PQQ
